# Nano-elicitation strategy to improve specialized metabolite pathways in plant cell suspension culture

**DOI:** 10.3389/fpls.2025.1679901

**Published:** 2025-11-28

**Authors:** Kamran Ashraf, Qamar uz Zaman, Weihua Tang, Pei Jiang, Xun Wan, Khitev Yuri, Ali Mohsin, Meijin Guo

**Affiliations:** 1 State Key Laboratory of Bioreactor Engineering, East China University of Science and Technology, Shanghai, China; 2 Department of Environmental Sciences, The University of Lahore, Lahore, Pakistan; 3 Shanghai Morimatsu Pharmaceutical Equipment Engineering Co Ltd., Shanghai, China; 4 Institute of Environmental Engineering, People's Friendship University of Russia (RUDN University), Moscow, Russia

**Keywords:** amino acids, elicitation, hormone, metabolites, osmolytes, phenolics

## Abstract

Plant hormone-loaded nanoparticles (NPs) represent a novel class of materials with significant potential in plant cell culture, owing to their unique physico-chemical properties. The utilization of these hormone-loaded NPs as elicitors could enhance the production of bioactive compounds and boost antioxidant enzymatic activity in plant cell suspension cultures. Therefore, this study aimed to synthesize jasmonic acid (JA) loaded Fe_3_O_4_ NPs and evaluate their effects on the cell suspension culture of *Carthamus tinctorius* (safflower). The synthesized material was applied at various concentrations (10, 20, 40 and 80 mg L^-1^) to assess its impact on cell growth, physio-biochemical, antioxidative activities and specialized metabolites (SMs) of *C. tinctorius*. The results demonstrated that the addition of JA-loaded NPs significantly enhanced the total chlorophyll (70.37%), soluble protein (154.45%) and total phenolic contents (110.64%) of safflower compared to the control. A linear decrease in all reactive oxygen species (ROS) attributes, such as H_2_O_2_ (4.65%) and O_2_^-^ (22.81%), was observed as the NPs concentration in the culture media was increased to the T2 group (20 mg L^-1^). Maximum chlorogenic acid (CGAs) accumulation (43.76 mg g^-1^) was noted on 72 hours after elicitation, representing a 2.26-fold increase over the control group. Furthermore, amino acid profiling revealed substantial variations in the composition of all detected amino acids following treatment with JA-loaded Fe_3_O_4_ NPs. In summary, this strategy demonstrates potential for optimizing the production of antioxidant and bioactive metabolites, thereby offering a viable solution for the industrial scale production of high-quality safflower extracts.

## Graphical abstract

**Figure:**
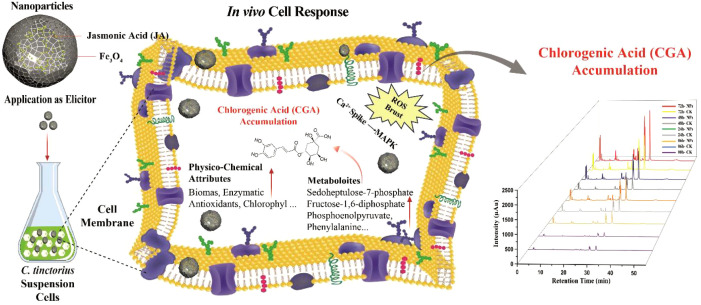

## Highlights

- Nano-elicitation is a novel technique for enhancing specialized metabolites.
- JA-loaded Fe_3_O_4_ NPs increased the chlorogenic acid accumulation to 2.26% compared to control.
- JA-loaded Fe_3_O_4_ NPs decrease the H_2_O_2_ and O_2_^-^ levels up to 4.65 and 22.81%, respectively.
- Sedoheptulose-7-phosphate, fructose-1, 6 diphosphate, and phosphoenolpyruvate enhanced the accumulation of aromatic amino acids.

## 1 Introduction

Cell suspension culture techniques represent a significant advancement as a scalable and well-controlled method to overcome the low yields of specialized metabolites (SMs), such as alkaloids, phenolics, and flavonoids in plant cells (Anuradha et al., 2025). The low yields present a major challenge for the large-scale extraction of these compounds for use in pharmaceuticals and nutraceuticals (Szopa et al., 2024). This technique simplifies the process of growing the plant cells in an optimized environment to encourage the production of desirable metabolites (Ashraf et al., 2025). Furthermore, it is a highly valuable method for inducing the biochemical pathways that lead to the production of specialized metabolites, a process in which elicitors act as key stimulating agents (Murthy et al., 2024; Xu and Xu, 2024). However, the application of conventional chemical elicitors is limited due to their low stability, poor bioavailability, and the need for repeated applications, which only produce transient effects (Xu and Xu, 2024). These drawbacks necessitate an immediate innovation in elicitation techniques that are not only more economical and sustainable but also promising to offer stable and high yielding effects (Imran et al., 2025).

The use of NPs for elicitation is an innovative and promising strategy to enhance the production of SMs in plant cells (Martínez-Chávez et al., 2024). The nano-sized dimension, high surface area to volume ratio, and enhanced reactivity of NPs provide desirable physico-chemical properties that facilitate the effective interaction with the plant cells (Duman et al., 2024; Kılıç et al., 2025). The most promising advantage is their capacity for the targeted delivery of elicitors directly to the plant’s metabolic machinery (Rani et al., 2025). This helps to overcome the limitations of conventional NPs-based elicitors in cell suspension cultures, which include premature degradation, uneven distribution, and transient exposure, all of which can lead to suboptimal metabolite induction and cellular stress (Mohammadinejad et al., 2019; Kalia and Sreelakshmi, 2025). Abiotic elicitors, including the NPs (Jeyasri et al., 2023), can regulate the specialized metabolites by activating the transcription of genes involved in the defense related biosynthetic pathways (Golkar et al., 2019; Ejaz et al., 2024). For instance, traditional elicitors have been shown to induce the synthesis of chlorogenic acid (CGAs) and related enzymatic antioxidants in safflower and other cell cultures (Haghighi and Majumdar, 2025). In this context, megantic NPs loaded with plant hormones emerge as an efficient and sustainable tool for enhancing the production of specialized metabolites in cell suspension cultures (Khan et al., 2025).

The unique properties of Fe_3_O_4_ NPs include high surface area, magnetism, and biocompatibility, all of which affect cellular processes (El-Khawaga et al., 2025). The application of these NPs enhances cell division and biomass growth by balancing the cellular environment (Wang et al., 2025). With regard to antioxidative potential, Fe_3_O_4_ NPs after uptake undergo Fenton like reactions, which induce the synthesis of reactive oxygen species (ROS) in a regulated way, resulting in the plant defense mechanisms activation (Kulus et al., 2025). The Fe_3_O_4_ NPs offer the controlled delivery of iron ions (Fe^2+^/Fe^3+^), which are essential co-factors for the enzymes in phenylpropanoid pathway (Ayoobi et al., 2024). Moreover, jasmonic acid (JA) also plays a significant role in regulating the overall performance of safflower cell suspension cultures, particularly in secondary metabolism (Samanta and Roychoudhury, 2025). It has been reported that JA in cell culture trigger the activity of key enzymes, including phenylalanine ammonia-lyase (PAL), chalcone synthase (CHS), and other downstream catalysts, which are effective in the metabolic flux towards the synthesis of specialized metabolites (Elbouzidi et al., 2024). The NPs facilitate the effective delivery of JA into plant cells, which is crucial for the JA signaling pathway. This pathway regulates major key enzymes that deal with the biosynthesis of SMs such as alkaloids, flavonoids, and terpenoids (Ravi et al., 2025). Due to the magnetic nature of Fe_3_O_4_, the distribution and accumulation of NPs in the culture can be controlled easily, which results in persistent and accelerated metabolite production (Yousaf et al., 2025). To date, there is limited literature on the use of JA-loaded Fe_3_O_4_ NPs for metabolic regulation in plants.

Safflower (*Carthamus tinctorius* L.) is a widely recognized medicinal plant, esteemed for valuable SMs, like chlorogenic acids (CGAs), flavonoids, and anthocyanins, which serve as useful substitutes for synthetic compounds (Golkar and Taghizadeh, 2018; Ashraf et al., 2025). The biosynthesis of these SMs in safflower is a complex process that involves intricate enzymatic pathways interacting with inherent genetic regulatory mechanisms. Moreover, this biosynthetic process may be initiated or enhanced by the presence of external elicitors (Krishnan et al., 2024; Wu et al., 2025). Chlorogenic acids are key groups of these metabolites, notable for their role in the scavenging of ROS and mitigating oxidative stress (Fu et al., 2015; Talukder et al., 2025). Recent perspectives suggest that combining traditional elicitors with novel technologies such as NPs-based delivery systems, could enable a more specific and sustained increase in CGA content and enhance the antioxidant profile in safflower (Sobhy et al., 2025). However, the mechanisms by which phytohormone-loaded nanomaterials affect CGA biosynthesis, antioxidant status, and ROS levels, remain unclear. Elucidating these processes is crucial for enhancing cellular CGA production. The main objectives of this study were to: 1) explore the structure and composition of the JA-loaded Fe_3_O_4_ NPs, 2) evaluate the variations in physio-biochemical attributes, enzymatic antioxidants, and ROS concentrations in safflower cells treated with different levels of JA-doped Fe_3_O_4_ NPs, and 3) assess the transcriptional expression of key biosynthetic genes. The outcomes of this research highlight an innovative approach for synthesizing JA-loaded Fe_3_O_4_ NPs to enhance biosynthesis of bioactive metabolites. This approach effectively modulates ROS and improves antioxidative systems. By establishing a connection between the elicited physio-biochemical responses and the transcriptional regulation of pivotal genes, this study provides a comprehensive and practical strategy for augmenting the accumulation of CGAs in *C. tinctorius*.

## 2 Materials and methods

### 2.1 Nanoparticles synthesis & characterization

#### 2.1.1 Preparation of carbon spheres

Carbon spheres were synthesized by dissolving 6 g glucose monohydrate in 40 mL of deionized water to form a clear solution. This solution was transferred to an oven-type autoclave with Teflon liner and heated in an oven at 180°C for 320 min. Subsequently, the autoclave was removed and allowed to cool naturally at room temperature (20°C). The resulting product was then centrifuged, and the precipitate of carbon spheres was washed several times using distilled water and absolute ethanol. Following a further centrifugation step at 8,000 rpm for 10 minutes, the material was dried in a vacuum oven at 60 °C for 480 min.

#### 2.1.2 Preparation of hollow nanospheres of Fe_3_O_4_

A 40 mmol amount of iron nitrate nanohydrate (Fe_3_(NO_3_)_3_.9H_2_O) was dissolved in a mixture of absolute ethanol and deionized water, following the methodology of our previous study (Liu et al., 2025). The mixture was sonicated for 30 minutes to ensure the thorough mixing. Subsequently, 0.6 g of urea was added, and the solution was stirred and sonicated for a further 30 minutes. After this, 200 mg of carbon spheres were introduced, and the mixture was stirred for another 30 minutes. The solution was then sonicated for 15 minutes and heated in an oil bath at 90°C for 360 minutes. The resulting material was centrifuged, washed with absolute ethanol and deionized water, and dried in an oven at 60°C. Finally, the product was calcined at 450°C to obtain hollow structure iron oxide.

#### 2.1.3 Synthesis of jasmonic acid loaded Fe_3_O_4_

The NPs suspension was prepared by dissolving 500 mg of NPs in 50 mL of ultrapure water. Subsequently, 50 mg of jasmonic acid (JA) [Sigma-Aldrich (Shanghai) Trading Co., Ltd., Shanghai, China] was added to this suspension. The mixture was incubated for 48 hours at room temperature on an orbital shaker. Following this, the Ja-loaded NPs were collected by centrifugation (12,000 × g rpm, 15min, and 18°C) and the resulting pellets were stored at 4°C.

#### 2.1.4 Jasmonic acid loaded Fe_3_O_4_ nanoparticles characterization

The surface morphology and the crystal structure were determined using an X-ray diffraction (XRD) (XTrA ARL, Switzerland) with Cu Kα radiation operated at 30 kV and scanning rate of 10°/min. The X-ray spectroscopy (EDS) TEM consisted of JEM-2100 CX instrument (JEOL Ltd., Japan running at 200 kV) with which the internal structure of the material was evaluated. X-ray photoelectron spectroscopy (XPS) was performed to analyze the surface chemical composition and elemental states of the material. The estimation of the elemental and functional groups was conducted for all NPs in the XPS performed on the PHI 5000 Versa probe (monochromatic Al source) by ULVAC-PHI Ltd., Japan. The binding energy of all samples was calibrated by fixing the C1s photoelectron peak at 284.6eV, and the data was analyzed using XPS Peak 4.1 program. Nitrogen adsorption-desorption isotherm at 77K on ASAP 2010 (Micromeritics Company, USA) was used to calculate the BET surface area and distribution of pore size of the material. All the samples were subjected to zeta potential-pH profiling in order to establish their isoelectric point (IEP). The measurements were conducted at 20°C, wavelength: 658 nm, power: 40 mW with the photon correlation spectroscopy under the Litesizer 500 apparatus of the Anton Paar GmbH, Austria. All analyses were performed in triplicate to ensure precision.

### 2.2 Elicitation of *C. tinctorius* cells

Safflower, belonging to the family Asteraceae (Compositae), was collected from Yining county, Xinjiang province, China, following authentication by a certified botanist. The identification of the plant was based on its morphological characteristics, which include ovate leaves, orange-red tubular florets, and a prominent taproot. A suspension cell culture system was established utilizing the callus derived from the sepals, as documented in previous studies (Liu et al., 2023; Ashraf et al., 2025). For the experiments, cells were harvested by filtration, ground in liquid nitrogen, and used for total RNA extraction. The callus material was inoculated at 4 g fresh weight into 40 mL of B5 liquid medium, representing a 10% of (*m/v*) inoculum size. The cultures were grown in the dark on a rotary shaker at 28°C and 115 rpm (0.37 × g and 28°C) for six days (Liu et al., 2024), with subculturing performed every 10 days. Prior to experimentation, the cultured cells were sequentially filtered through 12–40 mesh sieves to remove cell clumps and extracellular metabolic by-products (Liu et al., 2023). For the treatment, cells cultured for three days were subjected to co-culturing for further three days with various concentrations (0, 10, 20, 40 and 80 mg L^-1^) of JA-loaded Fe_3_O_4_ NPs. Harvesting the cells from the cultures was repeated every 24 hours to analyze the cellular metabolite levels. The B5 medium includes [basal salt of B5, 6-benzylaminopurine, sucrose and α-naphthaleneacetic acid at 3.21 g L^-1^, 2 × 10^−4^ g L^-1^, 30 g L^-1^, 4 × 10^−4^ g L^-1^], respectively, all supplied by “Qingdao Hope Bio-Technology Co., Ltd” in China.

### 2.3 Growth attributes of cultured cells

The growth dynamics of the cell suspension cultures were assessed by measuring the fresh biomass in seven days old cultures, using an electric weighing balance. For each measurement, three replicates were analyzed. Subsequently, the cultured cells were harvested, oven-dried at 50°C, and the dry cell biomass was determined.

### 2.4 Pigment and osmolyte attributes

A sample of approximately 0.4 g was vortexed in 10 mL of 80% acetone. The solution was then filtered and centrifuged at 5000 × g for 5 min at 25°C. The absorbance of the filtrate was measured at 644.8 and 661.8 nm and 470 nm as quantities of chlorophyll a and b and carotenoids, respectively. The total chlorophyll content was calculated by adding the chlorophyll a and b. The Coomassie brilliant blue G250 was used to determine the level of soluble protein (Bradford, 1976). Approximately 0.1g of sample was homogenized with 5 mL of phosphate buffer at pH 7.8. The homogenate was centrifuged at 4500 × g for 10 min. Subsequently, 0.9 mL of distilled water and 0.1 mL enzyme extract were added to 5 mL of Coomassie brilliant blue G250 reagent. The absorbance was recorded at wavelength of 595 nm.

### 2.5 Determination of enzymatic antioxidants and ROS related attributes

Enzyme activity assays were conducted using commercial kits (Solar Bio-Science & Technology (Beijing, China) Co., Ltd.) to determine the enzymatic antioxidants parameters in embryogenic callus. The activities of these enzymatic antioxidants were assayed according to manufacturer protocols. One unit of the enzyme activity was defined as the change in absorbance per minute per gram of tissue per milliliter of the reaction system, measured at a specific wavelength.

O_2_^-^ concentrations were quantified according to the method of Bu et al. (2016). A 0.1 g sample of cell balls-up was homogenized with 2 mL 50 mM phosphate buffer (pH 7.8) and centrifuged at 10,000 × g for 20 minutes. Then, 0.5 mL of the resulting extraction solution was incubated with 0.5 mL of 50 mM phosphate buffer (pH 7.8) and 1.5 mL of 1 mM hydroxylamine hydrochloride at 25°C for 1 hour. Subsequently, 2 mL of 17 mM p-aminobenzene sulfonic acid and 2 mL of 7 mM 2-naphthylamine were added to the reaction mixture for incubation at 25°C for 20 minutes, and the absorbance was measured at 530 nm. A method used by Bu et al. (2016) was followed to calculate H_2_O_2_ concentration. A 0.1 g cell sample was processed with 1 mL ice-cold acetone and centrifuged at 3000 × g for 10 minutes. To the extracted supernatant, 0.1 mL of 5% (*w/v*) titanous sulfate and 0.2 mL ammonia were added, followed by 0.1 mL hydrochloric acid. This solution was centrifuged at 3000 × g for 10 minutes. The pellet was then dissolved in 5 mL of 2 M sulfuric acid, and the absorbance of the final solution was measured at 410 nm.

### 2.6 Determination of antioxidant profile and total phenolics

The antioxidant potential of safflower extract cells was evaluated by determining its radical-scavenging activities of ABTS and DPPH assays alongside the ferric reducing antioxidant power (FRAP) following the established procedure (Sethi et al., 2020; Zhao et al., 2020). Furthermore, the total phenolic content (TPC) was quantified by the folin-Ciocalteu method (Orak et al., 2015). The results of TPC were reported in g equivalent catechin per g extract.

### 2.7 Detection of intracellular metabolites

The determination of CGAs in cells was carried out by subjecting intracellular extracts to an assay using a 1260 HPLC system from Agilent Technologies, USA. The mobile phase consisted of 0.05% (v/v) phosphoric acid solution as component (A) and acetonitrile as component (B). The gradient elution was as follows: from 0 to 40 minutes, 5–41% B; from 40 to 42 minutes, 41–90% B; from 42 to 45 minutes, 90–5% B; and from 45 to 55 minutes, maintained at 5% B. These procedures were conducted following the protocols established by Liu et al. (2024). The gas chromatography-mass spectrometry (GS-MS) chromatograph coupled with an Agilent 5977B mass selective detector and a DB-5MS capillary column (30 m × 0.25 mm i.d., 0.25 µm film thickness) was used to analyze intracellular metabolite concentrations. The quenching, extraction, derivatization, and detection of these primary metabolites were performed in accordance with previous studies (Liu et al., 2024; Chen et al., 2020).

### 2.8 Statistical analysis

The experiment was designed using a completely randomized design (CRD) with three replicates for each treatment. The analysis of variance (ANOVA) and the treatment means were compared using the Tukey’s Honest Significant Difference (HSD) test at the 5% significance level. Statistical analyses were performed using the Statistics 8.01 software, while multivariate analyses were executed with the R Studio software package.

## 3 Results

### 3.1 Material characterization

#### 3.1.1 Scanning electron microscopy

**Figure 1:**
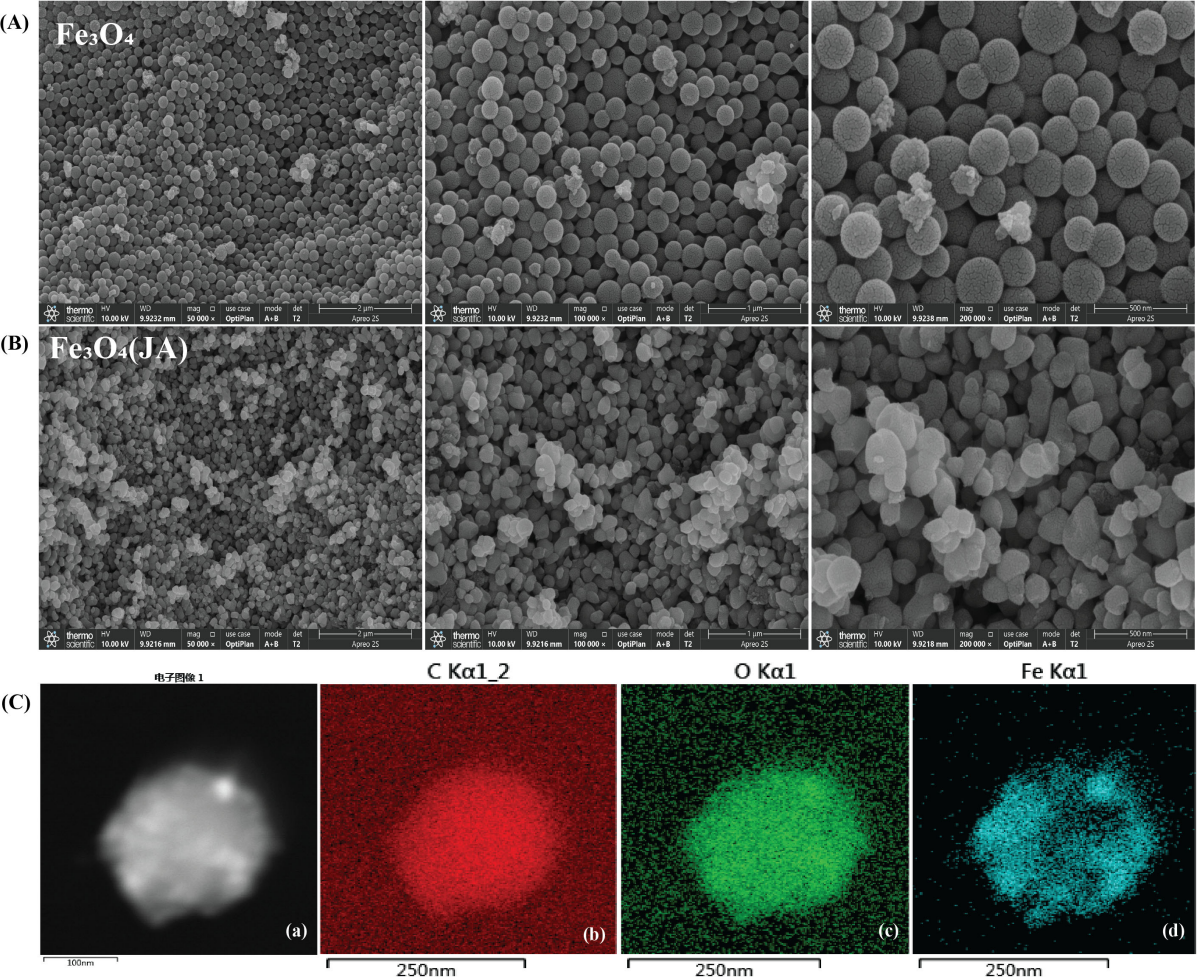
SEM analysis of synthesized material at different magnifications, **(A)** hollow spherical structure of Fe_3_O_4_ NPs; **(B)** Fe_3_O_4_ NPs loaded with JA; **(C, **a**)** EDS of aggregated NPs; (b) EDS-TEM of C, (c) EDS-TEM of O_2_, (d) EDS-TEM of Fe.

**Figure 1** presents scanning electron microscopy (SEM) and transmission electron microscopy (TEM) images of the synthesized magnetic NPs at different magnifications. The Fe_3_O_4_ particles exhibited a distinct spherical shape, as illustrated in **Figure 1A**, with minor agglomeration noted during synthesis. The particle size distribution was relatively consistent, spanning 100–200 nm, with distinct hollow spherical characteristics observable at higher magnification. These features suggest that the particles were synthesized in a controlled manner, exhibiting uniform diameters and consistent distribution. The Jasmonic acid (JA) loaded Fe_2_O_3_ NPs depicted in **Figure 1B** exhibit a relatively uniform and well-defined morphology, reflecting effective surface modification and minimal aggregation. The regular shape and smooth surface texture suggest a stable and successful interaction between JA and the Fe_3_O_4_ nanostructures. The observed morphological alterations, characterized by spherical forms and particles distributed in the range of 100 to 250 nm, indicate the successful loading of JA (Joshi et al., 2022).

#### 3.1.2 Transmission electron microscopy

To examine the finer structural details at nanoscale levels and to obtain crystallographic/phase information, TEM analysis was conducted subsequent to SEM. The EDS-TEM analysis in **Figure 1C** confirmed the presence of carbon, oxygen, and iron, with a uniform elemental distribution across the hollow spherical Fe_3_O_4_ NPs. The strong carbon signal supports the successful loading of JA, while the iron and oxygen signatures validate the presence of Fe_3_O_4_, consistent with the reference (Sohail et al., 2020). These results confirm the successful synthesis and functionalization of hollow Fe_3_O_4_ NPs, highlighting their potential for future biological and environmental applications.

#### 3.1.3 X-diffraction

**Figure 2:**
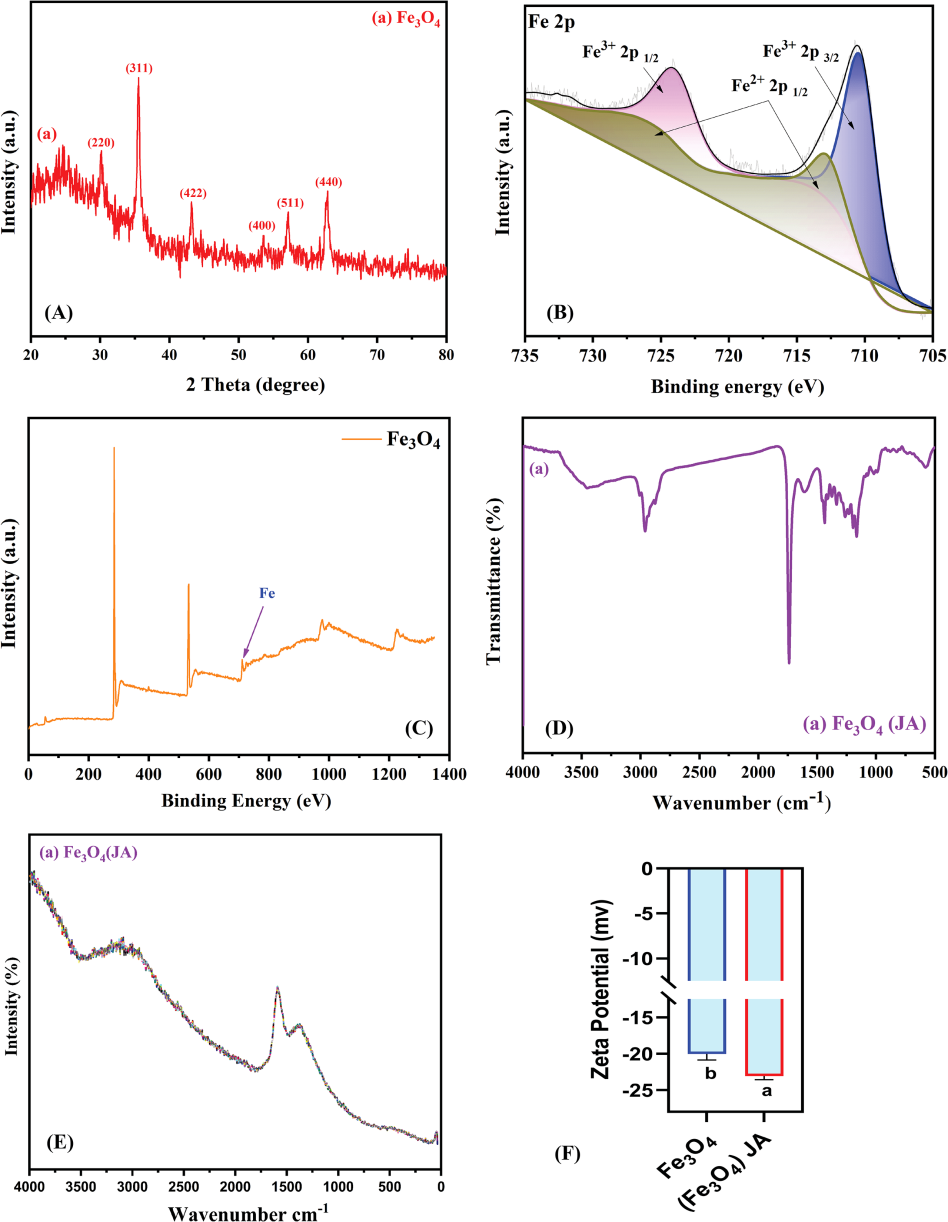
**(A)** XRD pattern of Fe_3_O_4_ magnetic NPs; **(B)** XPS spectra of Fe_3_O_4_ magnetic NPs; **(C)** XPS full spectra of Fe_3_O_4_**(D)** FTIR spectra of JA-loaded Fe_3_O_4_; **(E)** Raman spectra of magnetic JA-loaded Fe_3_O_4_ NPs; **(F)** Zeta potential measurement; The results were reported as the mean values + standard deviation (n = 3). Letters above bars showed significant differences (at 5% level) based on a Tukey’s-HSD test.

To confirm the bulk crystal structure and phase purity of the material, XRD analysis was conducted following TEM analysis, as the latter provides only localized nanoscale information. **Figure 2** depicts the structural and surface chemical assessment of Fe_3_O_4_ magnetic NPs. The XRD pattern in **Figure 2A** shows a distinct diffraction peaks at 2θ values of approximately 30.1° (220), 35.4° (311), 43.1° (400), 53.5° (422), 57.0° (511), and 62.6° (440), corresponding to the crystallographic planes of the cubic spinel structure of Fe_3_O_4_, which is consistent with the literature (Sobhannizadeh et al., 2025). The sharp, well-defined nature of the peaks indicates the high crystallinity of the synthesized NPs.

#### 3.1.4 X-ray photoelectron spectroscopy

To determine the elemental composition and chemical states of the surface, XPS analysis was conducted following XRD, as it provides surface-specific chemical information, crucial for understanding material properties and reactivity. The XPS spectra in **Figures 2B, C** offer deep insights into the surface oxidation states of iron in the synthesized material. The sharp peaks evident at binding energies of ~ 70.18 eV are assigned to Fe 2p 3/2 and Fe 2p 1/2, respectively, while the satellite peak at ~ 713.3 eV is characteristic of Fe 2p 3/2 and Fe 2p 1/2. The confirmed presence of both Fe^2+^ and Fe^3+^ confirmed the mixed-valence state nature of Fe_3_O_4_, indicating the successful synthesis of magnetic NPs, which is consistent with previous literature reports (Kasote et al., 2019).

#### 3.1.5 Fourier transform infrared spectroscopy

FTIR analysis complements XPS by identifying molecular functional groups and bonding vibrations. The spectrum in **Figure 2D** displays distinctive absorption bands representative of both Fe_3_O_4_ and JA moieties. A broad band at approximately 3400 cm^-1^ corresponds to O-H stretching vibrations, whilst the peak near 1630 cm^-1^ is ascribed to the C=O stretching of carboxyl groups in JA. Furthermore, bands observed below 700 cm^-1^ are indicative of Fe-O vibrations, confirming the integrity of the magnetic Fe_3_O_4_ core following the surface modification (Nasrollahzadeh et al., 2021).

#### 3.1.6 Raman spectral analysis

Raman analysis complements FTIR by detecting non-IR-active vibrational modes and providing details on molecular symmetry. The Raman spectral data in **Figure 2E** further supported the structural features of the synthesized NPs. The high intensity peak in the low wavenumber area was attributed to the A_1_g vibrational mode of magnetite. The minimal shifts and variations in peak intensities suggest surface interactions between Fe_3_O_4_ core and JA, thereby proving the successful functionalization.

#### 3.1.7 Zeta potential analysis

Zeta potential analysis is essential for assessing colloidal stability and surface charge to gain a comprehensive understanding of material properties. As exhibited in **Figure 2F**, the loading of JA induced a significant change. The Zeta potential of the pure Fe_3_O_4_ NPs was -20 mV, which decreased to about -15 mV for the JA-loaded Fe_3_O_4_ NPs. This change suggests a reduction in surface charge density and indicates that the organic JA coating enhanced the colloidal stability of the NPs (Meng et al., 2024).

#### 3.1.8 Brunauer-Emmett-Teller analysis

To determine surface area and porosity, BET analysis is a necessary complement after Zeta potential. Together, these techniques provide insights into NPs’ surface chemistry (from Zeta potential) and physical structure (from BET), which are essential for evaluating their suitability for applications such as catalysis and adsorption. **Table 1** displays the BET-analysis results for Fe_3_O_4_ and JA-loaded Fe_3_O_4_ NPs. Loading with JA led to a significant increase in both surface area (from 7.802 to 12.352 m²/g) and average pore size (from 6.616 to 8.205 nm), although a minor reduction in total pore volume was observed (Meng et al., 2024). These alterations suggest an improvement in surface properties, likely resulting from the organic loading.

**Table 1:** BET specific surface area, pore size distribution and pore volume.

Sample	BET/m^2^/g	Pore size/nm	Pore volume/cm^3^/g
Fe_3_O_4_	7.802 ± 0.037 ^b^	6.616 ± 0.027^c^	0.029 ± 0.028^b^
(Fe_3_O_4_) JA	12.352 ± 0.101^a^	8.205 ± 0.022 ^a^	0.027 ± 0.011^a^

The differences between the treatments were considered to be significant and were marked in the columns with lower case letters.

### 3.2 Application of synthesized nano elicitors with safflower cell suspension culturing and its bioactive defense molecules changes

#### 3.2.1 Biomass attributes

**Figure 3:**
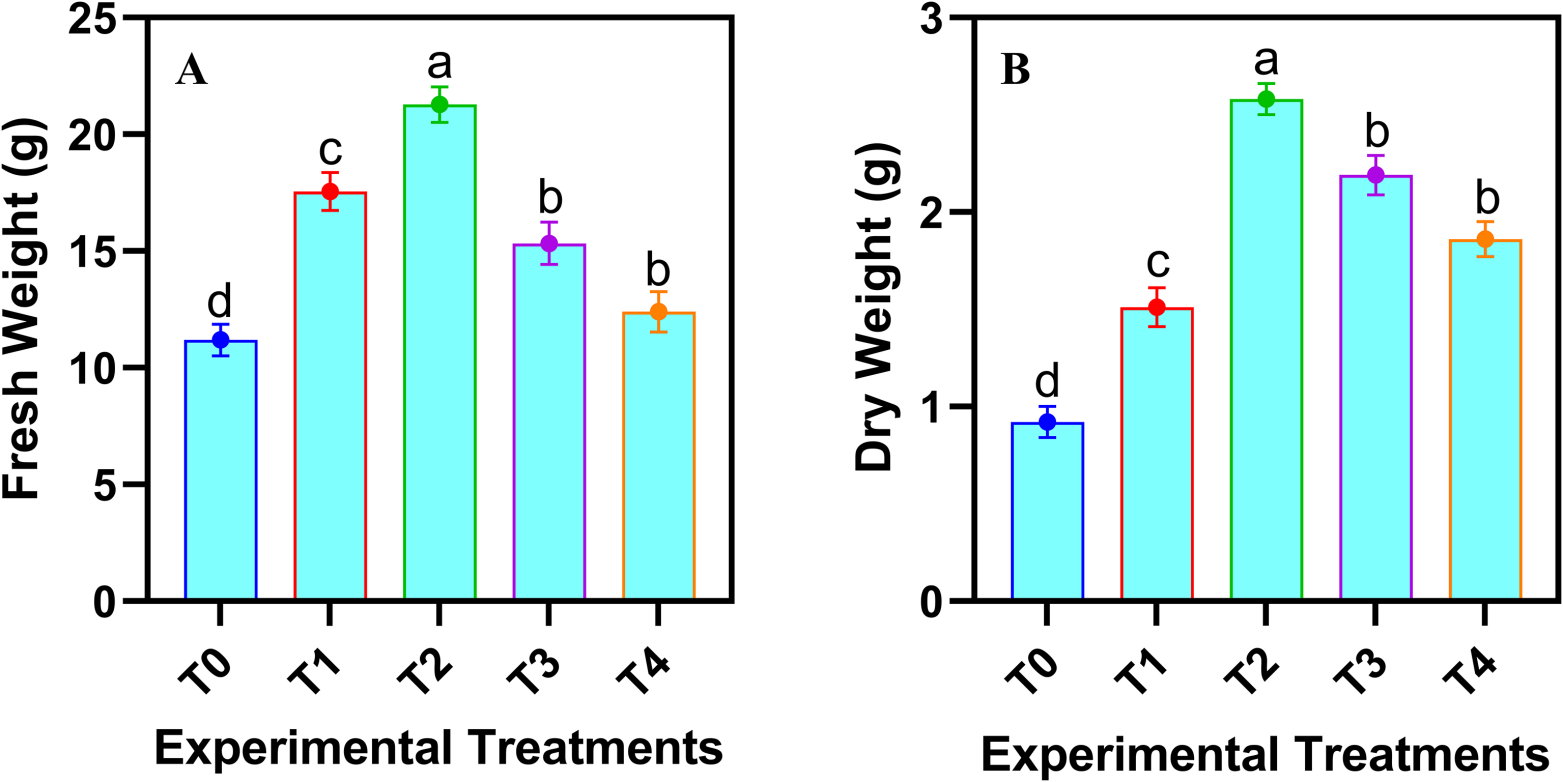
Effects of various doses of JA loaded Fe_3_O_4_ NPs on **(A)** fresh weight of cells; **(B)** dry weight of safflower cells in suspension culture. The results were reported as the mean values + standard deviation (n = 3). Letters above bars showed significant differences (at 5% level) based on a Tukey’s-HSD test. T_0_ = 0 mg L^-1^, T_1_ = 10 mg L^-1^, T_2_ = 20 mg L^-1^, T_3_ = 40 mg L^-1^ and T_4_ = 80 mg L^-1^.

The fresh and dry biomass of safflower cells in the suspension culture were significantly (*p* ≤ 0.05) affected by the various levels of JA-loaded Fe_3_O_4_ NPs (**Figure 3**). Both fresh and dry biomass increased linearly up to a concentration of 20 mg L^-1^, beyond which a decline was observed maximum increase in cell fresh weight (76.63%) and dry weight (149.51%), was observed by the application of 20 mg L^-1^ of JA-loaded Fe_3_O_4_ NPs in the cell suspension culture of safflower, relative to control group (0 mg L^-1^).

#### 3.2.2 Pigment and osmolyte attributes

**Figure 4:**
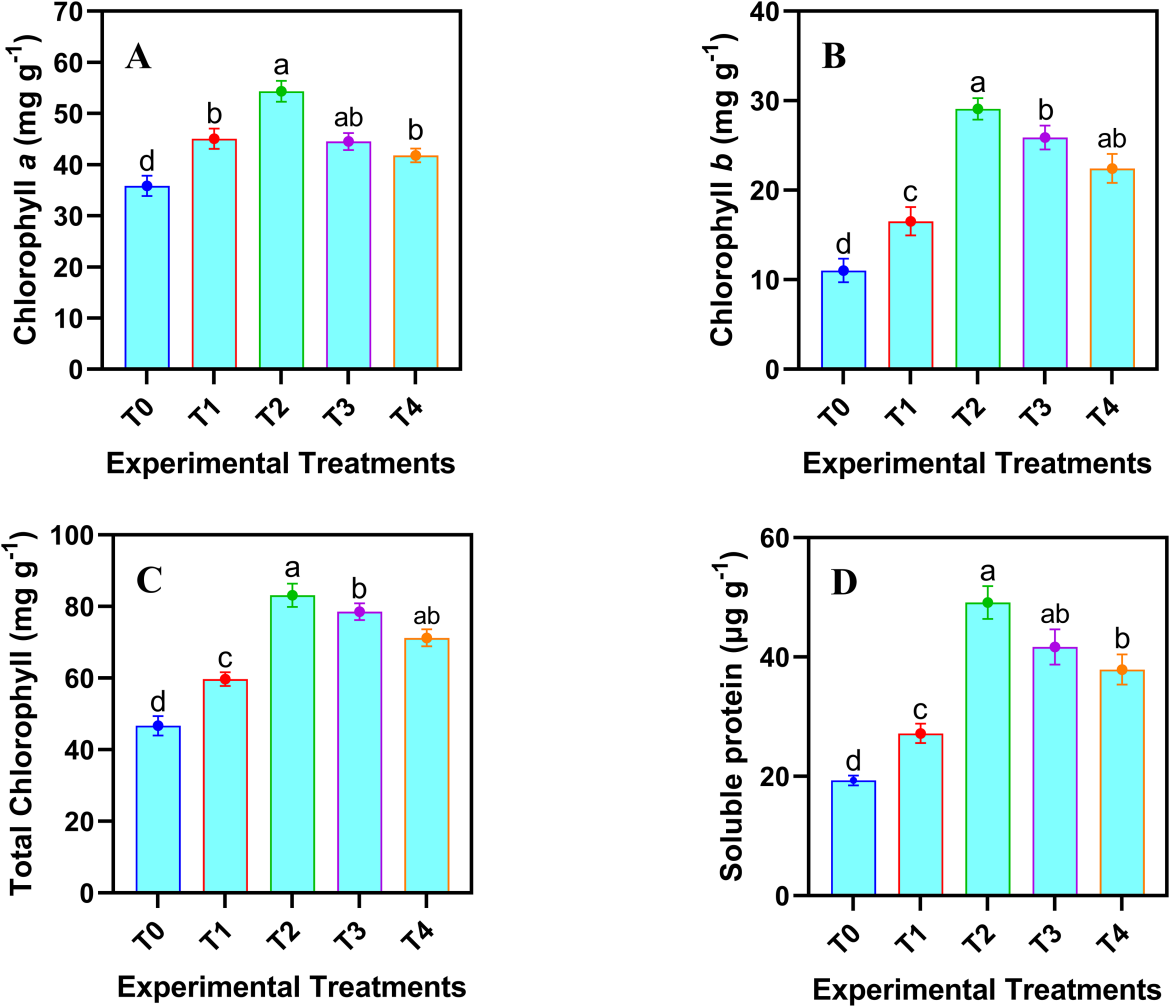
Effects of various doses of JA loaded Fe_3_O_4_ NPs on **(A-C)** chlorophyll (*a*, *b*, Total) contents; **(D)** soluble protein in safflower cells in suspension culture. The results were reported as the mean values + standard deviation (n = 3). Letters above bars showed significant differences (at 5% level) based on a Tukey’s-HSD test. T_0_ = 0 mg L^-1^, T_1_ = 10 mg L^-1^, T_2_ = 20 mg L^-1^, T_3_ = 40 mg L^-1^ and T_4_ = 80 mg L^-1^.

The results in **Figure 4** exhibited that application of various levels of JA-loaded Fe_3_O_4_ NPs significantly (*p* ≤ 0.05) affected the pigment and osmolyte attributes of safflower cells in the suspension culture. Maximum increase in chlorophyll a (46.24%), chlorophyll b (150.30%), total chlorophyll (70.37%) and soluble protein (154.45%) was observed by the application of 20 mg L^-1^ of JA-loaded Fe_3_O_4_ NPs, relative to the control group (0 mg L^-1^).

#### 3.2.3 Enzymatic antioxidants and ROS related attributes

**Figure 5:**
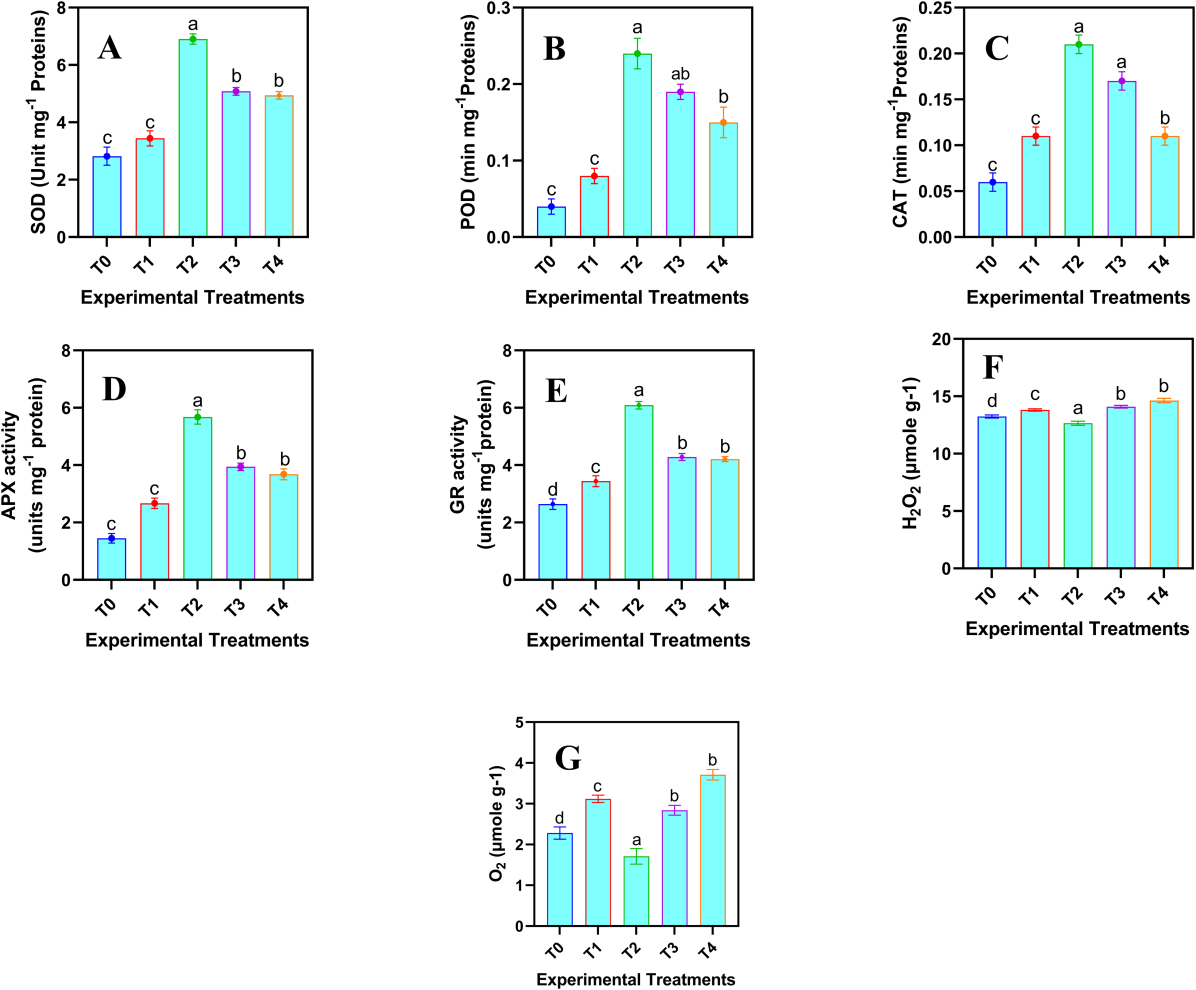
Effects of various doses of JA loaded Fe_3_O_4_ (µM) NPs on the enzymatic antioxidants and ROS related attributes in safflower in suspension culture. **(A)** superoxide dismutase activity (SOD); **(B)** peroxidase activity (POD); **(C)** catalase activity (CAT); **(D)** ascorbate peroxidase activity (APX); **(E)** glutathione reductase activity (GR) **(F)** H_2_O_2_ contents; **(G)** O_2_^-^ contents. The results were reported as the mean values + standard deviation (n = 3). Letters above bars showed significant differences (at 5% level) based on a Tukey’s-HSD test. T_0_ = 0 mg L^-1^, T_1_ = 10 mg L^-1^, T_2_ = 20 mg L^-1^, T_3_ = 40 mg L^-1^ and T_4_ = 80 mg L^-1^.

To assess the plant’s oxidative stress response and detoxification capacity for mitigating ROS generated under stress, the quantification of enzymatic antioxidants is essential. This provides a comprehensive understanding of plant defense mechanisms. The enzymatic antioxidant and ROS related attributes of safflower cells in the suspension culture were significantly (*p* ≤ 0.05) affected by the various levels of JA-loaded Fe_3_O_4_ NPs (**Figure 5**). A linear increase in the enzymatic antioxidants was observed up to 20 mg L^-1^; however, at the highest concentration (80 mg L^-1^), a decline in all the activities of enzymatic antioxidants was observed. Maximum values of SOD activity (6.90 ± 0.18 Unit mg^-1^ protein), POD activity (0.24 ± 0.02 min mg^-1^ protein), CAT activity (0.21 ± 0.01 min mg^-1^ protein), APX activity (5.68 ± 0.25 Unit mg^-1^ protein), and GR activity (6.09 ± 0.13 Unit mg^-1^ protein) were observed where 20 mg L^-1^ of JA loaded Fe_3_O_4_ NPs were added in the cell suspension culture of safflower, compared to control. Similarly, the optimal level of JA-loaded Fe_3_O_4_ NPs decreased the H_2_O_2_ (4.65%) and O_2_^-^ (22.81%) as compared with the control group.

#### 3.2.4 Total phenolic contents

**Figure 6:**
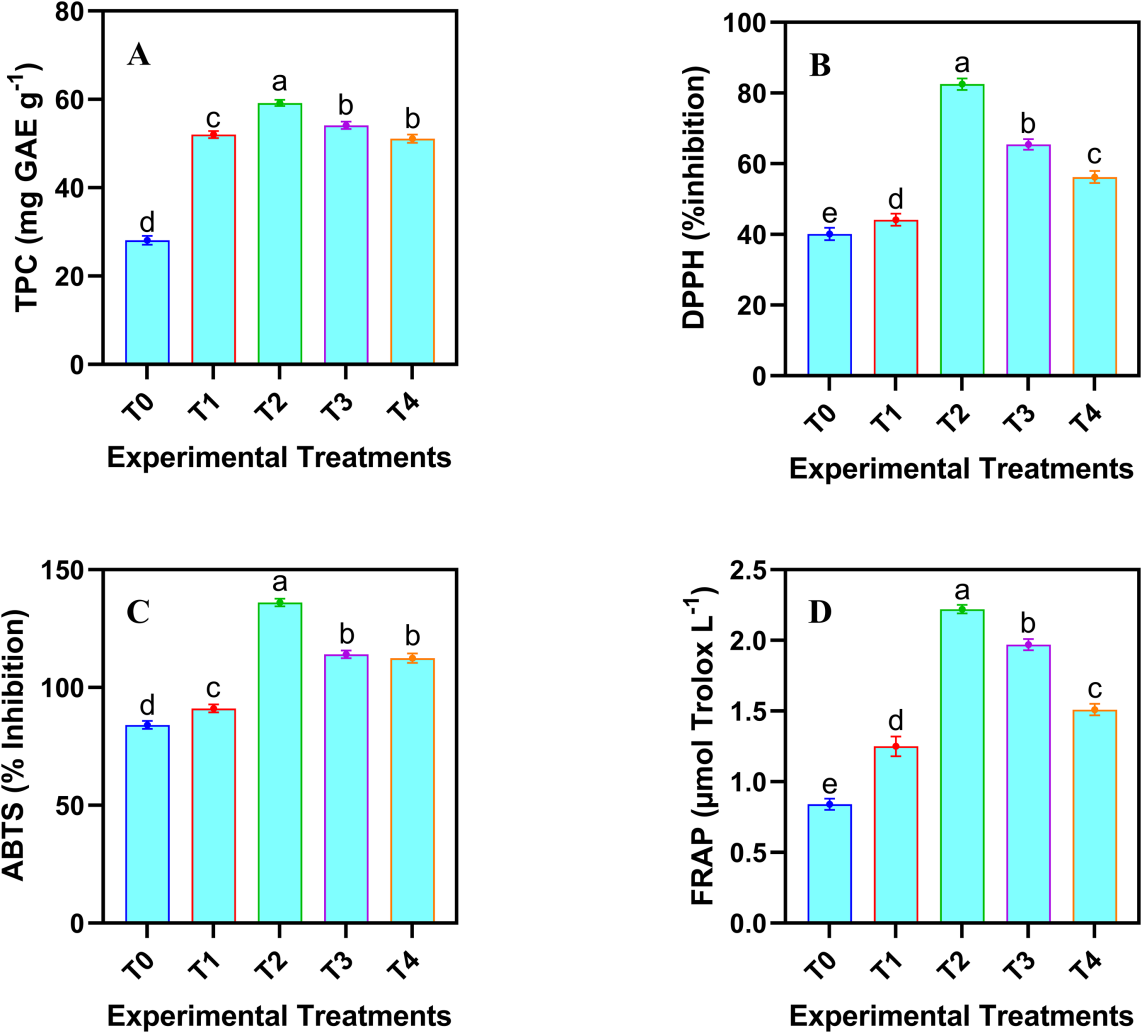
Effects of different doses of JA loaded Fe_3_O_4_ NPs on phenolics conetents and antioxidant profile in safflower cells in suspension culture. **(A)** total phenolic contents; **(B)** DPPH scavenging activity; **(C)** ABTS activity; **(D)** FRAP activity. The results were reported as the mean values + standard deviation (n = 3). Letters above bars showed significant differences (at 5% level) based on a Tukey’s-HSD test. T_0_ = 0 mg L^-1^, T_1_ = 10 mg L^-1^, T_2_ = 20 mg L^-1^, T_3_ = 40 mg L^-1^ and T_4_ = 80 mg L^-1^.

The analysis of phenolic contents provides a comprehensive view of non-enzymatic defense mechanisms, ensuring a holistic assessment of the plant’s antioxidant capacity. A dose-dependent increase in the total phenolics was observed in the safflower cell suspension culture following the application JA-loaded Fe_3_O_4_ NPs (**Figure 6**). Maximum increase in the total phenolic contents (110.64%) was noticed under the T2 treatment group. However, the highest concentration of JA-loaded Fe_3_O_4_ NPs decreased the total phenolics compared to the control and T_2_ treatment group.

#### 3.2.5 Antioxidant profile

Antioxidant profiling confirmed the functional activity of phenolics by demonstrating their efficacy in neutralizing ROS. The application of JA-loaded Fe_3_O_4_ NPs significantly (*p* ≤ 0.05) improved the antioxidant capacity of cultured cells (**Figure 6**). Various concentrations of JA-loaded Fe_3_O_4_ NPs produced a linear increase in the antioxidant activity, as measured by ABTS, DPPH, and FRAP assays. However, this trend reversed at higher concentrations (40 and 80 mg L^-1^), which resulted in a reduced antioxidant profile. The maximum increase in the percent inhibition was observed when 20 mg L^-1^ of JA-loaded Fe_3_O_4_ NPs was treated compared to the control. This indicates that the maximum level of JA-loaded Fe_3_O_4_ NPs induced a decline in the antioxidant activity across all the methods tested.

#### 3.2.6 Accumulation of chlorogenic acid

**Figure 7:**
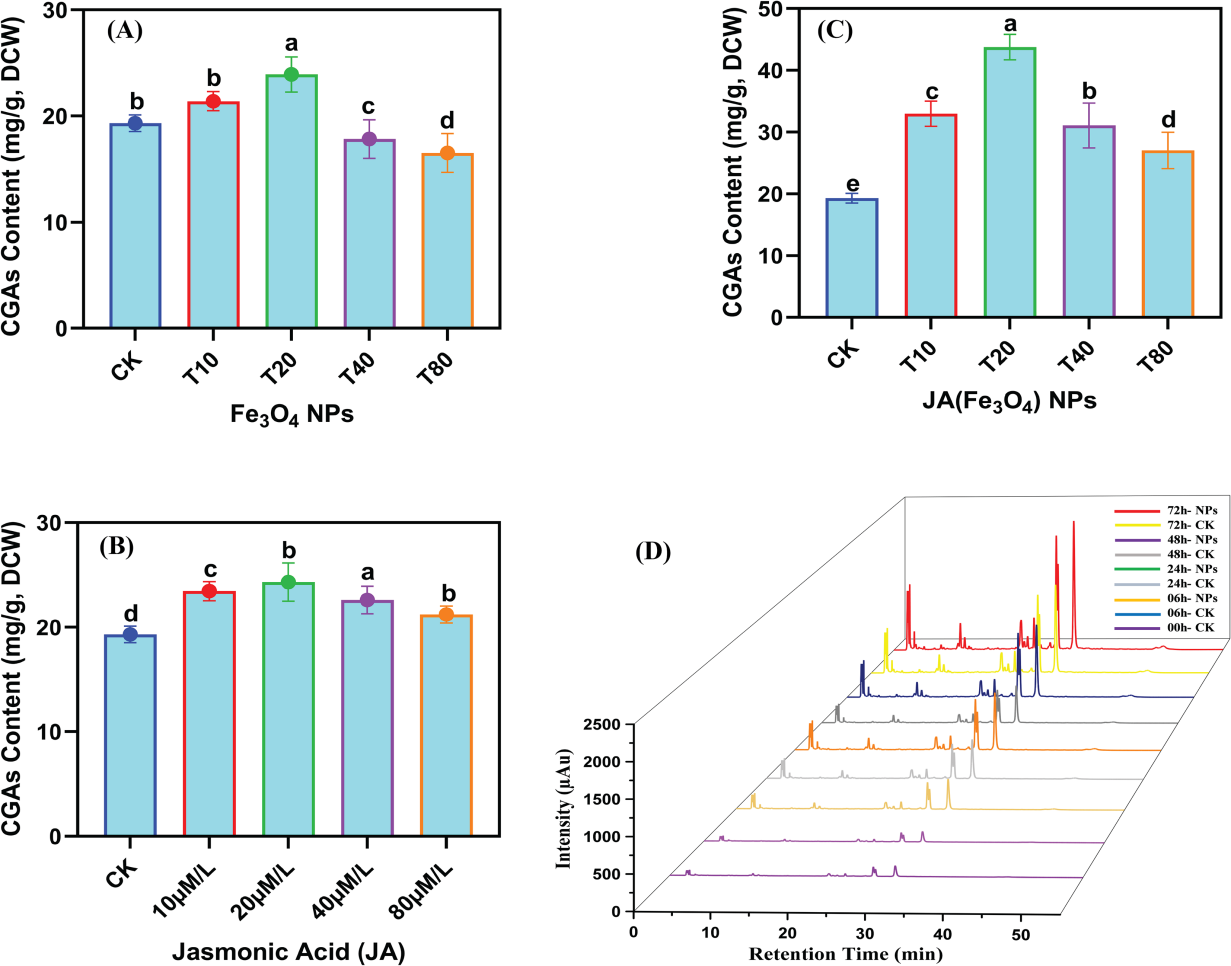
Temporal regulation of JA-loaded Fe_3_O_4_ NPs on the CGAs accumulation in *C*. *tinctorius* cells determined through high performance liquid chromatography; dose responsive CGAs accumulation in *C*. *tinctorius* cells **(A)** by Fe_3_O_4_ NPs; **(B)** by Jasmonic Acid; **(C)** Jasmonic acid loaded Fe_3_O_4_; **(D)** Temporal variation of CGAs accumulation in *C*. *tinctorius* cells. Bars with different letters are significantly different (5%) based on a Tukey’s-HSD test; CK = 0 mg L^-1^; T10 = 10 mg L^-1^; T20 = 20 mg L^-1^; T40 = 40 mg L^-1^; T80 = 80 mg L^-1^.

The measurement of CGAs contents, following the assessment of antioxidant traits, helped to confirm its contributions to total antioxidant activity. When various concentrations of Fe_3_O_4_, JA, and JA-loaded Fe_3_O_4_ NPs were added to *C. tinctorius* cell cultures, an increase in CGAs content in cells was observed, followed by a subsequent decline (**Figures 7A-C**). This pattern indicates that both JA and Fe_3_O_4_ NPs induce the biosynthesis of CGAs. The highest CGAs contents detected were 23.91 mg g^-1^ in the group treated with 20 mg L^-1^ Fe_3_O_4_ NPs and 24.31 mg g^-1^ in the group treated with 20 µM JA. Notably, the protocol for enhancing the CGA production was significantly more effective when JA was loaded into the Fe_3_O_4_, showing a positive dose-dependent effect. The highest CGAs contents were found in the T2 treatment group (i.e., 43.76 mg g^-1^ by 20 mg L^-1^ JA-loaded Fe_3_O_4_ NPs-treated) compared to the control, which is 2.44-fold higher than the control. This identifies 20 mg L^-1^ JA-loaded Fe_3_O_4_ NPs as the optimal treatment condition. The chromatogram in **Figure 7D**, which plots retention time against signal intensity of differentially treated samples over time, shows the concentration of CGAs at 0, 6, 24, 48 and 72 hours. A significant enhancement in overall CGA concentration was seen in the NPs-treated group as the co-culture period extended from 06–72 h. The highest cumulative CGA content was observed in 72 hours of elicitation (43.76 mg g^-1^), which is 2.26 times that of the control group. This confirms that CGA accumulation was highly augmented by elicitation with JA-loaded Fe_3_O_4_ NPs.

#### 3.2.7 Alterations in the primary metabolites profile

**Figure 8:**
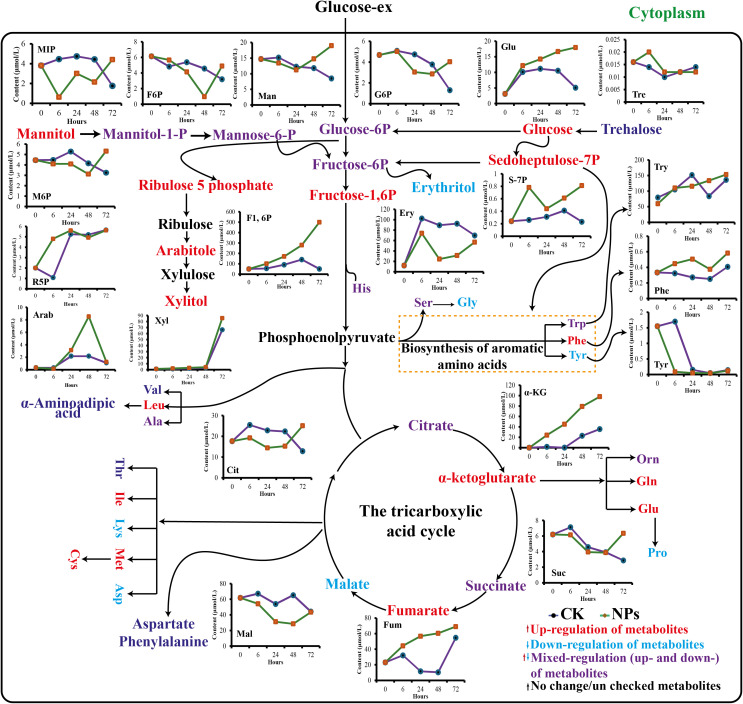
Temporal changes in metabolites production from synthesis of amino acid pathways in NPs treated cells.

Primary metabolites, such as amino acids, are essential precursors and energy sources for producing specialized metabolites. In this study, we analyzed 21 amino acids in cells treated with and without NPs (**Figure 8**). The results revealed substantial variance in the composition of all detected amino acids. Several amino acids, including Leucine, Valine, aspartate, Proline, Lysine and Glycine were generally down-regulated. In contrast, six other Phenylalanine, tyrosine, Glutamine, Glucose, Cystine and tryptophan showed a consistent upward trend (**Supplementary Figure S1**, **Supplementary Material**). It has been reported that the continual upregulation of molecules such as sedoheptulose-7-phosphate, fructose-1,6-diphosphate, and phosphoenolpyruvate increased the accumulation of aromatic amino acids, particularly phenylalanine, which is a critical CGA precursor (Liu et al., 2024). The up-regulation of phenylalanine observed in this study is significant because high levels of this amino acid can directly increase the substrate pool for the enzyme phenylalanine ammonia-lyase (PAL). This phenomenon facilitates the diversion of additional carbon into the phenylpropanoid pathway, thereby supporting the biosynthesis of CGA. Other up-regulated amino acids, such as tyrosine, along with alanine and glutamate, which participate in nitrogen metabolism (Feduraev et al., 2020), and transamination can further modulate this pathway by influencing the energy status, redox balance, and the supply of amino donors for biosynthesis (Yang et al., 2020). Consequently, the long-term NPs treatment appears to stimulate primary metabolism, promoting the elevated levels of precursors vital for the enhanced accumulation of CGA (Fu and Jiang, 2025).

#### 3.2.8 Regression analysis

**Figure 9:**
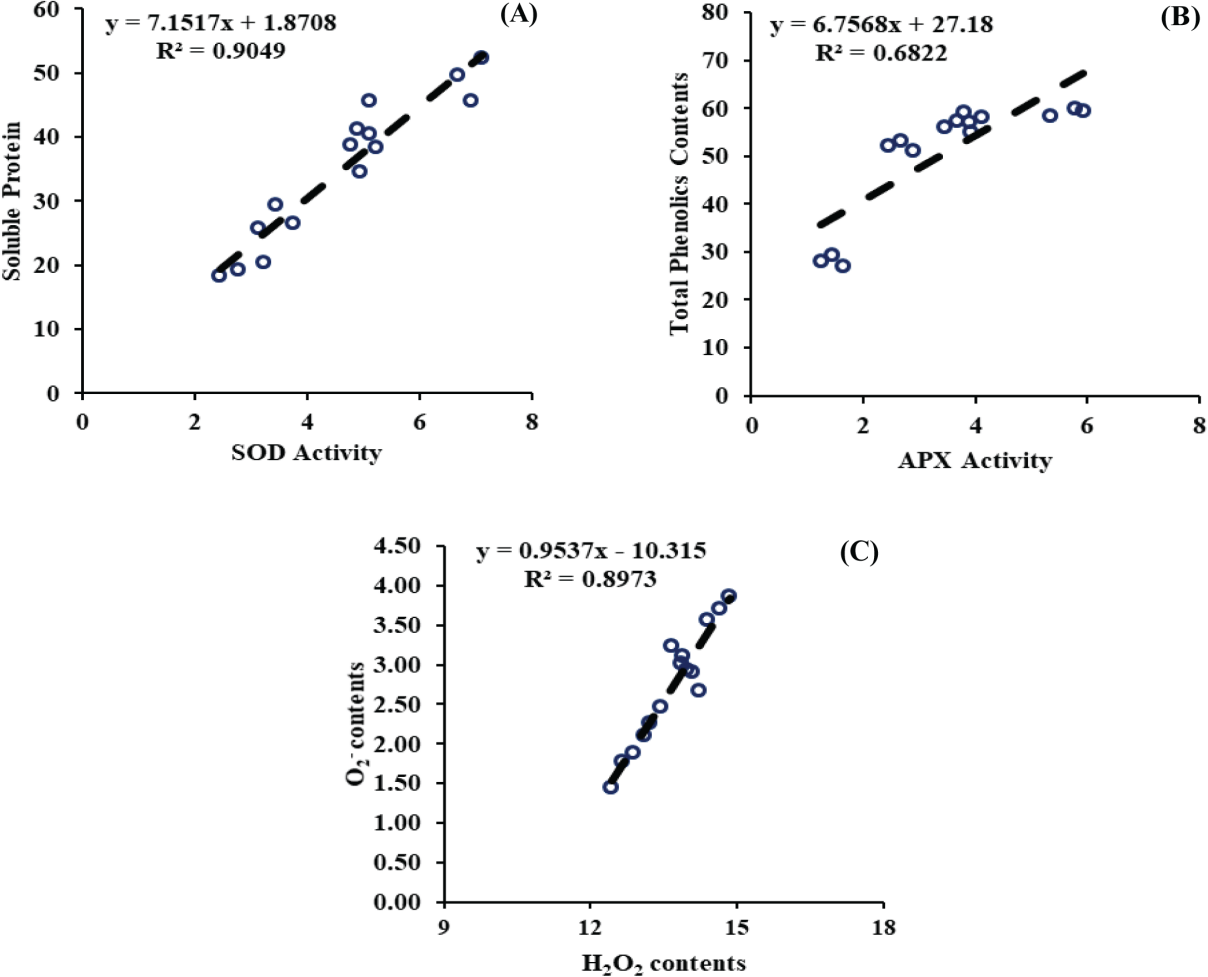
**(A)** Regression analysis between soluble protein and superoxide dismustase activity, **(B)** Regression analysis between total phenolics contents and ascorbate peroxidase activity, **(C)** Regression analysis between hydrogen peroxide and O2-

A simple linear regression analysis was performed to evaluate the relationship between the measured attributes. The model indicated a strong relationship, R^2^ value of soluble protein and SOD activity (R^2^ = 0.9049), between the total phenolics and APX activity (R^2^ = 0.68.22), and between the H_2_O_2_ contents and O_2_^-^ (0.8973), indicating a strong relationship (**Figure 9**). These relationships were statistically significant at the 95% confidence level. These findings suggest that optimizing the application rates of JA-loaded Fe_3_O_4_ NPs can significantly improve the enzymatic antioxidants by minimizing the impacts of ROS-related attributes.

#### 3.2.9 Multivariate and response surface methodology analysis

**Figure 10:**
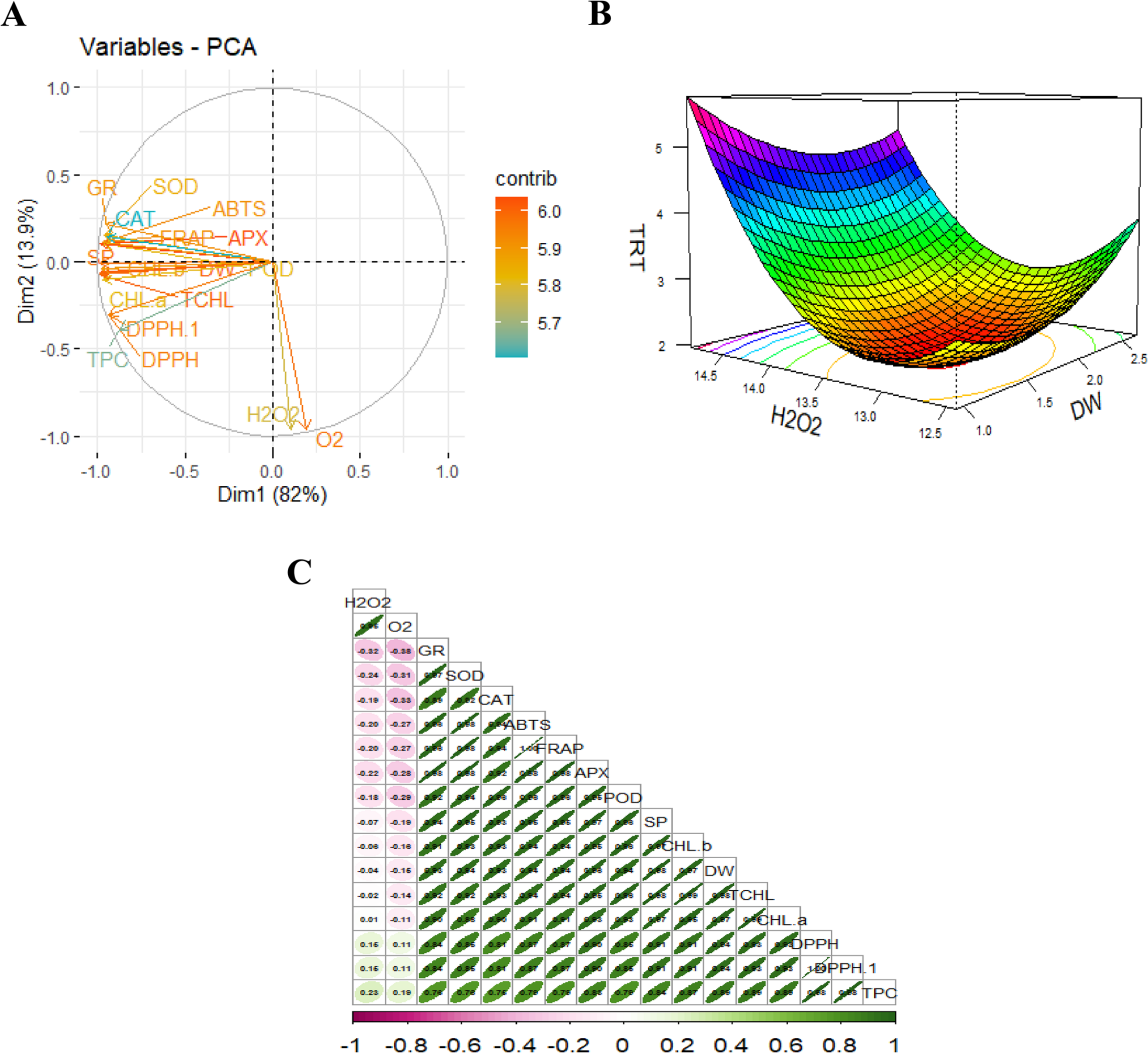
Multivariate analysis of measured parameters under various levels of JA-loaded Fe_3_O_4_ NPs; **(A)** Principal Component Analysis (PCA) biplot showing variable contributions; **(B)** 3D response surface model illustrating the interaction between key variables; **(C)** Correlation matrix highlighting relationships among measured parameters; T_0_ = 0 mg L^-1^, T_1_ = 10 mg L^-1^, T_2_ = 20 mg L^-1^, T_3_ = 40 mg L^-1^ and T_4_ = 80 mg L^-1^; FW, fresh weight of cells; DW, dry weight of cells; CHL, total chlorophyl contents; DPPH, (2,2-diphenyl-1-picrylhydrazyl) assay; SP, soluble protein contents; SOD, superoxide dismutase activity; POD, peroxidase activity; APX, ascorbate peroxidase activity; GR, glutathione reductase activity; TPC, total phenolics contents; H_2_O_2_, hydrogen peroxide activity and O_2_, reactive oxygen contents; ABTS = 2,2’-azino-bis (3-ethylbenzothiazoline-6-sulfonic acid).

To evaluate the relationship of measure attributes under the influence of various dosages of JA-loaded Fe_3_O_4_ NPs depicted in **Figures 10A-C**. The principal component analysis (PCA) explains a total variability of 82.00% for PC1 and 13.90% for PC2 components. The T_3_ cluster is in response to the relatively higher concentration of JA-loaded Fe_3_O_4,_ consisting of a group of enzymatic antioxidants associated as part of the plant’s defense mechanism. The T_4_ cluster represented the highest-level treatment applied (dose level~ 80 mg L^-1^), which primarily influenced total chlorophyll content, and fresh and dry weights of the safflower suspension cells (**Figure 10A**). Correlation analysis revealed a significant positive association among all biomass, chlorophyll and phenolics and antioxidative profiles. Conversely, the ROS-related attributes displayed a significant negative correlation with all the measured attributes (**Figure 10C**). Response surface methodology analysis confirmed that an increase in H_2_O_2_ levels corresponds with a decrease in dry weight, demonstrating a significant inverse relationship between the ROS and dry weight of safflower cells. Thus, treatments that upregulate also modulate H_2_O_2_ also modulate the cellular dry weight. The contour lines at the base of the plot further identify the optimal regions for achieving maximum DW content under moderate to high H_2_O_2_ and treatment conditions. Ultimately, this analyses underscore the principal contributions of H_2_O_2_ content and treatment optimization on biomass, highlighting the potential elicitor-based approaches to enhance the antioxidative and therapeutic properties of safflower cells.

## 4 Discussion

The study revealed the potential of JA-loaded Fe_3_O_4_ NPs for enhancing specialized metabolite biosynthesis in the *C. tinctorius*. Application of NPs, particularly magnetic NPs, in plant biotechnology has attracted significant interest due to their ability to enhance cellular uptake, target specific pathways (Holghoomi and Colagar, 2024). The JA is a plant hormone known to regulate numerous metabolic processes, making it an ideal candidate for improving the synthesis of specialized metabolites (Roychowdhury et al., 2025). Combining nanotechnology with plant regulators such as JA presents a promising strategy for advancing plant-based bio-manufacturing (Jadhav and Khare, 2024), offering a sustainable approach to boost the commercial production of specialized metabolites in plant cells.

The results demonstrate that the optimal concentration of JA-loaded Fe_3_O_4_ NPs improved the cell biomass, pigment attributes and soluble protein accumulation (**Figure 3**). The Fe_3_O_4_ NPs facilitated the efficient delivery of JA to the target cells, enhancing its uptake and the subsequent activation of signaling pathways (Ayoobi et al., 2024). The activation of these pathways may have increased the photosynthetic activity, directly leading to the observed higher chlorophyll concentration (Bi et al., 2024), which might be the possible reason for the improved pigment attributes in the cells. The application of Fe_3_O_4_ NPs probably contributed to enhanced nutrient uptake and cellular respiration (Zhang et al., 2025), which could explain the possible reason for higher biomass accumulation. The rise in soluble protein content suggests that JA-loaded NPs induced the expression of proteins associated with stress responses, development, and energy reserves (Ahmed et al., 2024). Furthermore, the NPs may have alleviated oxidative stress by scavenging free radicals, thereby helping to maintain cellular integrity and functioning. The synergistic effect of JA and Fe_3_O_4_ NPs appears to have enhanced key cellular functions, thereby promoting healthier overall growth and metabolism.

The optimal dose of JA-loaded Fe_3_O_4_ NPs enhanced the activity of critical antioxidant enzymes in the *C. tinctorius*, thereby increasing enzymatic antioxidants levels (**Figure 5**). This aligns with the findings of Dong (2025), who reported that an enhanced antioxidant mechanism effectively neutralizes ROS, alleviating oxidative stress and protecting cellular integrity. The NPs facilitated the sustained release of JA, which further enhanced the production of antioxidant response (Singh et al., 2025). It is probable that the interaction of JA and Fe_3_O_4_ NPs activated signaling pathways leading to the expression of antioxidant genes (Sobhannizadeh et al., 2025). This process reduced ROS accumulation and helped to regulate the cellular redox homeostasis (Li et al., 2025), thereby preventing the oxidative damage to lipids, proteins, and DNA. The consequent reduction in oxidative traits, such as protein carbonylation, certifies increased cell viability (Nègre-Salvayre and Salvayre, 2024). The treatment induced a balanced elevation of antioxidants (enzymatic and non-enzymatic), which might be the possible reason for the observed decrease in oxidative damage. Furthermore, the ROS are known to interact with the defense hormones, like JA, and the efficient delivery of JA was likely potentiated by the magnetic properties of Fe_3_O_4_ (Haghpanah et al., 2025). Enzymatic antioxidants, which consist of protein-based structures, facilitate the conversion of ROS into less reactive compounds (Haghpanah et al., 2024). For instance, superoxide dismutase (SOD) catalyzes the dismutation of superoxide (O_2_−) into hydrogen peroxide (H_2_O_2_) and molecular oxygen (O_2_). In a similar manner, catalase (CAT) detoxifies hydrogen peroxide by decomposing it into water and oxygen. Moreover, JA role in modulating cellular processes was potentiated by the magnetic properties of Fe_3_O_4_ NPs, ensuring efficient delivery and action of the hormone within the cells (Sobhannizadeh et al., 2025; Kumar et al., 2025). This synergistic interaction between JA and Fe_3_O_4_ NPs likely contributed to the improved stress resilience and physiological performance observed in the cell suspension culture.

The optimal concentration of JA-loaded Fe_3_O_4_ (20 mg L^-1^) significantly enhanced both antioxidant capacity and phenolic compound levels in *C. tinctorius*. Correlation and PCA revealed a positive association between the improved antioxidant activity and the JA-loaded Fe_3_O_4_ treatment. This can be explained by the NPs improving the bioavailability and the cellular uptake of JA. The enhanced interaction between the NPs and plant cellular structures likely increased the production of SMs (e.g., phenolic compounds), which contribute to antioxidant defense (Prasad et al., 2024). The PCA analysis further revealed the significant clustering of the treated samples, indicating that the JA-loaded Fe_3_O_4_ induce metabolic shift towards phenolic biosynthesis. However, at higher concentrations, the engineered NPs caused the dissolution of iron ions, elevating ROS (Xie et al., 2025) as supported by our H_2_O_2_ and MDA results. This oxidative damage likely inhibited the fundamental enzymatic functions and may have activated regulated cell death, ultimately suppressing secondary metabolic functions. This mechanism could be linked to the interaction of the Fe_3_O_4_ NPs with plant receptors or signaling pathways, triggering the production of phenolic metabolites as part of the plant’s stress-related responses (Saini et al., 2024).

**Figure 11:**
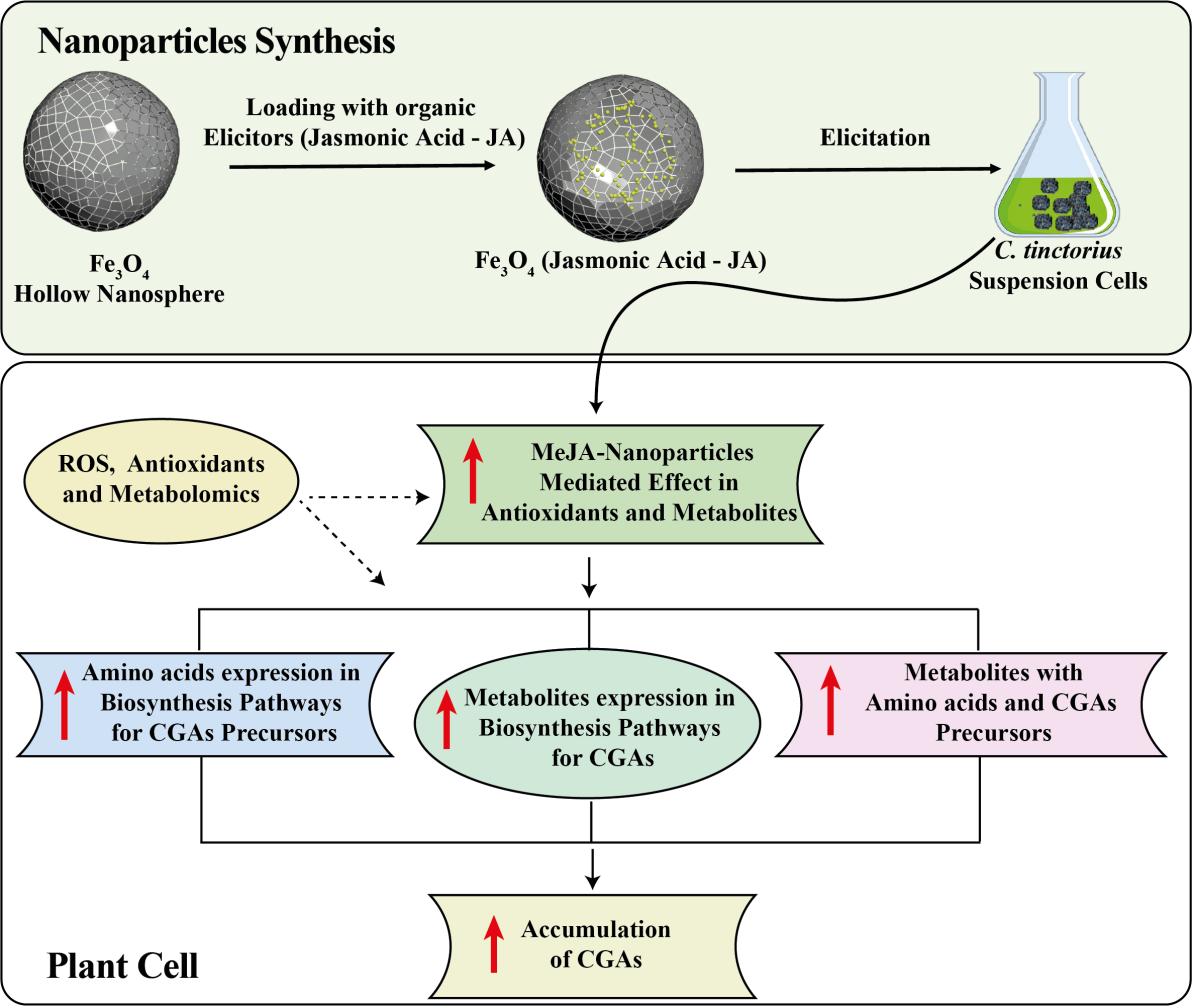
Mechanism diagram of Ja-loaded Fe_3_O_4_ NPs modulating the biosynthesis of CGAs in *C. tinctorius* cells.

The optimal concentration of JA-loaded Fe_3_O_4_ NPs significantly increased the production of primary metabolites in the *C. tinctorius* by modulating cellular metabolism (**Figure 11**). The iron based NPs facilitated the efficient delivery of JA to the cells, providing targeted action and reducing the hormone degradation (Yang et al., 2024). The JA stimulated the major biosynthetic pathways of the plant, resulting in up-regulated biosynthesis of CGAs (Lv et al., 2024) that are regarded to have antioxidant functions (Ashraf et al., 2025; Liu et al., 2024). The use of JA and interaction with Fe_3_O_4_ NPs also increased the nutrient and SMs uptake, which positively affects overall cell growth and metabolism (Ayoobi et al., 2024; Gjureci et al., 2025). Acting as a carrier, the NPs prevented the rapid breakdown of JA, ensuring the sustained release that prolonged its effects. This improved metabolic profile could be attributed to the synergistic interaction between JA signaling pathway modulation (Li et al., 2024) and the NPs’ physical properties, which enhanced cellular uptake and bioavailability. This combination increased the cell tolerance to ROS stress, a potential reason for the higher yield of the target metabolite. Furthermore, the NPs modulated the gene expression of major enzymes in metabolic pathways, thereby facilitating the synthesis of both primary and specialized metabolites (Liu et al., 2024; Ashraf et al., 2025; Singh et al., 2025). Increased resilience to oxidative stress and enhanced mitochondrial activity also contributed to the accumulation of CGAs and other metabolites (Thiruvengadam et al., 2024). The sustained release of JA by the NPs might be the possible reason for prolonged transcriptional activation, preventing feedback inhibition and effectively redirecting metabolic flux to improve the accumulation of CGAs. This enhanced biosynthetic capacity was correlated with the increased activity of transcription factors that regulate metabolism. These findings indicate that the plant hormone loaded magnetic NPs may be a useful tool for optimizing the metabolic profiles of the plants for biotechnological applications.

## 5 Conclusion

The optimum concentration of JA-loaded Fe_3_O_4_ NPs was observed to enhance cell growth and induce substantial increases in the antioxidative and phenolic content within the safflower cell suspension culture. Based on findings, the addition of 20 mg L^-1^ of the engineered NPs to the culture media enhanced the upregulation of antioxidative enzyme activities, total phenolic contents and CGA accumulation as indicated by the induction of an antioxidative defence system and the scavenging of ROS. This process highlighted an adaptive response involving the jasmonate production and its associated signaling pathways, which help to prevent oxidative stress and maintain cellular homeostasis in callus culture exposed to elicitors. The application of JA-loaded Fe_3_O_4_ has facilitated the development of a novel technique that significantly enhances the modulation of antioxidants in plant cell cultures. The work provides significant insights into the molecular pathways initiated by NP-based elicitors, positioning them as a promising tool for sustainable metabolic engineering and the large-scale biosynthesis of plant derived products. Further future investigations should include a larger sample size to facilitate comprehensive metabolite profiling and to quantify specific flavonoid variations, thereby elucidating the full phytochemical response on a wider scale. Additionally, conducting a time-course experiment is recommended to correlate JA release kinetics with the expression of CGA biosynthetic genes. This approach would clearly link the sustained JA signaling provided by NPs to the effective accumulation of CGA.

## Data Availability

The original contributions presented in the study are included in the article/**Supplementary Material**. Further inquiries can be directed to the corresponding authors.

## References

[B1] Ahmed M. Tóth Z. Decsi K. (2024). The impact of salinity on crop yields and the confrontational behavior of transcriptional regulators, nanoparticles, and antioxidant defensive mechanisms under stressful conditions: a review. Int. J. Mol. Sci. 25, 2654. doi: 10.3390/ijms25052654, PMID: 38473901 PMC10931947

[B2] Anuradha M. Balasubramanya S. Subbalakshmi G. Shilpa P. (2025). “ Commercialization of *in vitro* secondary metabolite production: Challenges and opportunities,” in *In Vitro* Production of Plant Secondary Metabolites: Theory and Practice, Springer, Singapore 321–346.

[B3] Ashraf K. Liu Z. Zaman Q. U. Arshad M. Zaman W. Q. Shan A. . (2025). *De novo* synthesis of selenium-doped CeO@Fe_3_O_4_ nanoparticles for improving secondary metabolite biosynthesis in *Carthamus tinctorius* cell suspension culture. Chem. Eng. J. 505, 159705. doi: 10.1016/j.cej.2025.159705

[B4] Ayoobi A. Saboora A. Asgarani E. Efferth T. (2024). Iron oxide nanoparticles (Fe_3_O_4_-NPs) elicited *Artemisia annua* L. *in vitro*, toward enhancing artemisinin production through overexpression of key genes in the terpenoids biosynthetic pathway and induction of oxidative stress. Plant Cell 156, 85. doi: 10.1007/s11240-024-02705-9

[B5] Bi X. Xu H. Yang C. Zhang H. Li W. Su W. . (2024). Investigating the influence of varied ratios of red and far-red light on lettuce (*Lactuca sativa*): effects on growth, photosynthetic characteristics and chlorophyll fluorescence. Front. Plant Sci. 15, 1430241. doi: 10.3389/fpls.2024.1430241, PMID: 39319008 PMC11419988

[B6] Bradford M. (1976). A rapid and sensitive method for the quantitation of microgram quantities of protein utilizing the principle of protein-dye binding. Anal. Biochem. 72, 248–254. doi: 10.1016/0003-2697(76)90527-3, PMID: 942051

[B7] Bu R. Xie J. Yu J. Liao W. Xiao X. Lv J. . (2016). Autotoxicity in cucumber (*Cucumis sativus* L.) seedlings is alleviated by silicon through an increase in the activity of antioxidant enzymes and by mitigating lipid peroxidation. J. Plant Biol. 59, 247–259. doi: 10.1007/s12374-016-0526-1

[B8] Chen J. Wang J. Wang R. Xian B. Ren C. Liu Q. . (2020). Integrated metabolomics and transcriptome analysis on flavonoid biosynthesis in safflower (*Carthamus tinctorius* L.) under MeJA treatment. BMC Plant Biol. 20, 353. doi: 10.1186/s12870-020-02554-6, PMID: 32727365 PMC7391820

[B9] Dong W. (2025). Synergistic effects of Fe_3_O_4_–NPs and *Enterobacter cloacae* in alleviating mercury stress in wheat (*Triticum aestivum* L.): insights into morpho–physio–biochemical attributes. Plant Physiol. Biochem. 223, 109881. doi: 10.1016/j.plaphy.2025.109881, PMID: 40188531

[B10] Duman H. Eker F. Akdaşçi E. Witkowska A. M. Bechelany M. Karav S. (2024). Silver nanoparticles: A comprehensive review of synthesis methods and chemical and physical properties. Nanomaterials 14, 1527. doi: 10.3390/nano14181527, PMID: 39330683 PMC11434896

[B11] Ejaz B. Mujib A. Syeed R. Mamgain J. Malik M. Q. Birat K. . (2024). Phytocompounds and regulation of flavonoids in *in vitro*-grown safflower plant tissue by abiotic elicitor CdCl2. Metabolites 14, 127. doi: 10.3390/metabo14020127, PMID: 38393019 PMC10891796

[B12] Elbouzidi A. Taibi M. Baraich A. Haddou M. Loukili E. H. Asehraou A. . (2024). Enhancing secondary metabolite production in Pelargonium graveolens Hort. cell cultures: Eliciting effects of chitosan and jasmonic acid on bioactive compound production. Horticulturae 10, 521. doi: 10.3390/horticulturae10050521

[B13] El-Khawaga A. M. Orlandini M. Raucci L. Elmaghraby K. (2025). Magnetic nanoparticles as a promising antimicrobial agent for combating multidrug resistant bacteria: A review. Discov. Appl. Sci. 7, 1–29. doi: 10.1007/s42452-025-06601-5

[B14] Feduraev P. Skrypnik L. Riabova A. Pungin A. Tokupova E. Maslennikov P. . (2020). Phenylalanine and tyrosine as exogenous precursors of wheat (*Triticum aestivum* L.) secondary metabolism through PAL-associated pathways. Plants 9, 476. doi: 10.3390/plants9040476, PMID: 32283640 PMC7238280

[B15] Fu D. Jiang B. (2025). Liquid-liquid phase separation regulates gene expression in plants. Agric. Commun. 3, 100084. doi: 10.1016/j.agrcom.2025.100084

[B16] Fu X. Yin Z. P. Chen J. G. Shangguan X. C. Wang X. Zhang Q. F. . (2015). Production of chlorogenic acid and its derivatives in hairy root cultures of Stevia rebaudiana. J. Agric. Food Chem. 63, 262–268. doi: 10.1021/jf504176r, PMID: 25548875

[B17] Gjureci B. Todorovska M. Stanoeva J. P. Tusevski O. Simic S. G. (2025). Elicitation of Hypericum perforatum L. hairy root cultures with salicylic acid and jasmonic acid enhances the production of phenolic compounds and naphthodianthrones with biological activities. Plant Cell Tissue Organ Culture (PCTOC) 160, 61. doi: 10.1007/s11240-025-03005-6

[B18] Golkar P. Taghizadeh M. (2018). *In vitro* evaluation of phenolic and osmolite compounds, ionic content, and antioxidant activity in safflower (*Carthamus tinctorius* L.) under salinity stress. Plant Cell Tissue Organ Culture (PCTOC) 134, 357–368. doi: 10.1007/s11240-018-1427-4

[B19] Golkar P. Taghizadeh M. Noormohammadi A. (2019). Effects of sodium alginate elicitation on secondary metabolites and antioxidant activity of safflower genotypes under *in vitro* salinity stress. In Vitro Cell. Dev. Biology-Plant 55, 527–538. doi: 10.1007/s11627-019-10008-4

[B20] Haghighi M. Majumdar S. (2025). “ The role of different elicitors in decreasing the deleterious effect of abiotic stress,” in Application of Eco-Friendly Exogenous Elicitors and Metabolic Dissection for Crop Improvement ( CRC Press, Boca Raton), 199–242.

[B21] Haghpanah M. Jelodar N. B. Zarrini H. N. Pakdin-Parizi A. Dehestani A. (2024). New Insights into Azelaic Acid-Induced Resistance against *Alternaria solani* in Tomato Plants. BMC Plant Biol. 24, 687. doi: 10.1186/s12870-024-05397-7, PMID: 39026164 PMC11264620

[B22] Haghpanah M. Namdari A. Kaleji M. K. Nikbakht-Dehkordi A. Arzani A. Araniti F. (2025). Interplay between ROS and hormones in plant defense against pathogens. Plants 14, 1297. doi: 10.3390/plants14091297, PMID: 40364326 PMC12073338

[B23] Holghoomi R. Colagar A. (2024). Applications of biocompatible nanoparticles in plant biotechnology for enhanced secondary metabolite biosynthesis. Inorg. Chem. Commun. 167, 112753. doi: 10.1016/j.inoche.2024.112753

[B24] Imran M. Feng X. Sun Z. Al Omari H. Zhang G. Zhu J. . (2025). Nanotechnology-driven gene silencing: advancements in SIGS–dsRNA technology for sustainable disease management. Chem. Biol. Technol. Agric. 12, 31. doi: 10.1186/s40538-025-00738-6

[B25] Jadhav R. R. Khare D. (2024). Green biotherapeutics: overcoming challenges in plant-based expression platforms. Plant Biotechnol. Rep. 18, 465–486. doi: 10.1007/s11816-024-00910-8

[B26] Jeyasri R. Muthuramalingam P. Karthick K. Shin H. Choi S. H. Ramesh M. (2023). Methyl jasmonate and salicylic acid as powerful elicitors for enhancing the production of secondary metabolites in medicinal plants: an updated review. Plant Cell Tissue Organ Culture (PCTOC) 153, 447–458. doi: 10.1007/s11240-023-02485-8, PMID: 37197003 PMC10026785

[B27] Joshi N. Pathak A. Upadhyaya D. C. Krishna S. B. N. Upadhyay C. P. (2022). Synthesis of biocompatible Fe_3_O_4_ and MnO_2_ nanoparticles for enhanced tuberization in potato (*Solanum tuberosum* L.). Biocatal. Agric. Biotechnol. 39, 102258. doi: 10.1016/j.bcab.2021.102258

[B28] Kalia A. Sreelakshmi M. V. (2025). “ Nanoelicitors: A promising strategy for sustainable crop production under biotic stress conditions,” in Elicitors for Sustainable Crop Production ( Springer, Singapore), 125–142.

[B29] Kasote D. M. Lee J. H. Jayaprakasha G. K. Patil B. S. (2019). Seed priming with iron oxide nanoparticles modulates antioxidant potential and defense-linked hormones in watermelon seedlings. ACS Sustain. Chem. Eng. 7, 5142–5151. doi: 10.1021/acssuschemeng.8b06013

[B30] Khan S. Zahoor M. Ullah R. Khan R. S. (2025). The uptake and mechanism of action of nanoparticles and doped nanoparticles on plant growth and metabolite enrichment. Environ. Technol. Rev. 14, 499–516. doi: 10.1080/21622515.2025.2508362

[B31] Kılıç G.Ç. Şeker M. G. Gutul T. Süzerer V. Dursun İ. Çiftçi Y. O. (2025). The influence of nanosized zero-valent iron (nZVI) on the micropropagation, antioxidant activity, and phenolic compound content of cherry laurel (*Prunus laurocerasus* L.). Plant Cell Tissue Organ Cult. 160, 68. doi: 10.1007/s11240-025-02968-w

[B32] Krishnan N. Singh P. K. Devadasan V. Mariappanadar V. Gopinath S. C. Chinni S. V. . (2024). Enhanced production of actinidine and glaziovine alkaloids from *Nardostachys jatamansi* (D. Don) DC. through cell suspension culture with elicitor treatment. Process Biochem. 138, 139–149. doi: 10.1016/j.procbio.2024.01.016

[B33] Kulus D. Tymoszuk A. Gościnna K. Osial M. (2025). Enhancing germination and growth of chrysanthemum synthetic seeds through iron oxide nanoparticles and indole-3-acetic acid: Impact of treatment duration on metabolic activity and genetic stability. Nanotechnol. Sci. Appl. 18, 139–155. doi: 10.2147/NSA.S503868, PMID: 40125333 PMC11929542

[B34] Kumar A. Partap M. Warghat A. R. (2025). Jasmonic acid: a versatile phytohormone regulating growth, physiology, and biochemical responses. J. Plant Growth Regul. 44, 131–154. doi: 10.1007/s00344-024-11376-x

[B35] Li Y. Jiang F. Niu L. Wang G. Yin J. Song X. . (2024). Synergistic regulation at physiological, transcriptional, and metabolic levels in tomato plants subjected to a combination of salt and heat stress. Plant J. 117, 1656–1675. doi: 10.1111/tpj.16580, PMID: 38055844

[B36] Li B. Ming H. Qin S. Nice E. C. Dong J. Du Z. . (2025). Redox regulation: mechanisms, biology and therapeutic targets in diseases. Signal Transduction Targeting Ther. 10, 72. doi: 10.1038/s41392-024-02095-6, PMID: 40050273 PMC11885647

[B37] Liu Z. Du L. Liu N. Mohsin A. Zhu X. Sun H. . (2023). Insights into chlorogenic acids' efficient biosynthesis through Carthamus tinctorius cell suspension cultures and their potential mechanism as α-glucosidase inhibitors. Ind. Crops Prod. 194, 116337. doi: 10.1016/j.indcrop.2023.116337

[B38] Liu Y. Gong S. Aldahmash W. Ashraf K. Li Z. Khan I. M. . (2025). Light-activated multimodal nanoplatform for enhanced synergistic therapy of breast cancer. Sci. Rep. 15, 25995. doi: 10.1038/s41598-025-11165-w, PMID: 40676066 PMC12271506

[B39] Liu Z. Zhu X. Mohsin A. Sun H. Du L. Yin Z. . (2024). Uncovering the role of hydroxycinnamoyl transferase in boosting chlorogenic acid accumulation in *Carthamus tinctorius* cells under methyl jasmonate elicitation. Int. J. Mol. Sci. 25, 2710. doi: 10.3390/ijms25052710, PMID: 38473957 PMC10931740

[B40] Lv L. L. Li L. Y. Xiao L. Q. Pi J. H. (2024). Transcriptomic and targeted metabolomic analyses provide insights into the flavonoids biosynthesis in the flowers of *Lonicera macranthoides*. BMC Biotechnol. 24, 19. doi: 10.1186/s12896-024-00846-5, PMID: 38609923 PMC11015657

[B41] Martínez-Chávez L. A. Hernández-Ramírez M. Y. Feregrino-Pérez A. A. Esquivel Escalante K. (2024). Cutting-edge strategies to enhance bioactive compound production in plants: potential value of integration of elicitation, metabolic engineering, and green nanotechnology. Agronomy 14, 2822. doi: 10.3390/agronomy14122822

[B42] Meng Z. Wu Q. Wu X. Yang C. Xu W. Lin T. . (2024). Nanoparticles of Fe_3_O_4_ loaded with azoxystrobin and pectin to enhance resistance of rice to sheath blight. ACS Appl. Nano Mater. 7, 2675–2686. doi: 10.1021/acsanm.3c04801

[B43] Mohammadinejad R. Shavandi A. Raie D. S. Sangeetha J. Soleimani M. Hajibehzad S. S. . (2019). Plant molecular farming: production of metallic nanoparticles and therapeutic proteins using green factories. Green Chem. 21, 1845–1865. doi: 10.1039/C9GC00335E

[B44] Murthy H. N. Joseph K. S. Paek K. Y. Park S. Y. (2024). Light as an elicitor for enhanced production of secondary metabolites in plant cell, tissue, and organ cultures. Plant Growth Regul. 104, 31–49. doi: 10.1007/s10725-024-01139-9

[B45] Nasrollahzadeh M. Shafiei N. Soleimani F. Nezafat Z. Bidgoli N. S. (2021). Physicochemical characterization of biopolymer-based metal nanoparticles. Biopolym. Met. Nanoparticle Chem. Sustain. Appl. 1, 317–478.

[B46] Nègre-Salvayre A. Salvayre R. (2024). Reactive carbonyl species and protein lipoxidation in atherogenesis. Antioxidants 13, 232. doi: 10.3390/antiox13020232, PMID: 38397830 PMC10886358

[B47] Orak H. H. Karamac M. Amarowicz R. (2015). Antioxidant activity of phenolic compounds of red bean (*Phaseolus vulgaris* L.). Oxid. Commun. 38, 67–76.

[B48] Prasad A. Sidhic J. Sarbadhikary P. Narayanankutty A. George S. George B. . (2024). Role of metal nanoparticles in organogenesis, secondary metabolite production and genetic transformation of plants under *in vitro* condition: a comprehensive review. Plant Cell Tissue Organ Cult. 158, 33. doi: 10.1007/s11240-024-02833-2

[B49] Rani N. Boora R. Singh Y. Choudhary Y. Devi S. Mohan N. . (2025). Nano-elicitation: An emerging and potential technique for enhanced production of pharmaceutically important secondary metabolites in plants. Nanotechnol. Environ. Eng. 10, 1–18. doi: 10.1007/s41204-025-00444-6

[B50] Ravi J. Ragunathan S. C. B. Mani S. Mythili R. Dixit S. Thayumanavan P. (2025). Biogenic fabrication of iron oxide nanoparticles using *Tinospora cordifolia* leaf extract: A green approach for enhanced antioxidant activity and bioadsorption, reusability, toxicity analysis of methylene blue dye. Luminescence 40, e70133. doi: 10.1002/bio.70133, PMID: 40032282

[B51] Roychowdhury R. Hada A. Biswas S. Mishra S. Prusty M. R. Das S. P. . (2025). Jasmonic acid (JA) in plant immune response: unraveling complex molecular mechanisms and networking of defence signalling against pathogens. J. Plant Growth Regul. 44, 89–114. doi: 10.1007/s00344-024-11264-4

[B52] Saini N. Anmol A. Kumar S. Wani A. Bakshi M. Dhiman Z. (2024). Exploring phenolic compounds as natural stress alleviators in plants— a comprehensive review. Physiol. Mol. Plant Pathol. 133, 102383. doi: 10.1016/j.pmpp.2024.102383

[B53] Samanta S. Roychoudhury A. (2025). Molecular crosstalk of jasmonate with major phytohormones and plant growth regulators during diverse stress responses. J. Plant Growth Regul. 44, 62–88. doi: 10.1007/s00344-024-11412-w

[B54] Sethi S. Joshi A. Arora B. Bhowmik A. Sharma R. Kumar P. (2020). Significance of FRAP, DPPH, and CUPRAC assays for antioxidant activity determination in apple fruit extracts. Eur. Food Res. Technol. 246, 591–598. doi: 10.1007/s00217-020-03432-z

[B55] Singh K. M. Jha A. B. Dubey R. S. Sharma P. (2025). Nanoparticle-mediated mitigation of salt stress-induced oxidative damage in plants: insights into signaling, gene expression, and antioxidant mechanisms. Environ. Sci. Nano 12, 2983–3017. doi: 10.1039/D5EN00174A

[B56] Sobhannizadeh A. Giglou M. T. Behnamian M. Estaji A. Majdi M. Szumny A. (2025). The effect of plant growth regulators, FeO_3_-CTs nanoparticles and LEDs light on the growth and biochemical compounds of black seed (*Nigella sativa* L.) callus in *vitro*. BMC Plant Biol. 25, 539. doi: 10.1186/s12870-025-06423-y, PMID: 40281420 PMC12032791

[B57] Sobhy S. E. Khalifa A. M. Hafez E. E. Elsherif D. E. (2025). Biosynthesized sulfur nanoparticles: a novel strategy to enhance antioxidant secondary metabolites in *Lotus arabicus* L. callus cultures. BMC Plant Biol. 25, 1–14. doi: 10.1186/s12870-025-06573-z, PMID: 40335942 PMC12057069

[B58] Sohail K. Kamran K. Kemmerling B. Shutaywi M. Mashwani Z. U. R. (2020). Nano zinc elicited biochemical characterization, nutritional assessment, antioxidant enzymes and fatty acid profiling of rapeseed. PloS One 15, e0241568. doi: 10.1371/journal.pone.0241568, PMID: 33170873 PMC7654759

[B59] Szopa A. Kwiecień I. Kubica P. Turcza-Kubica K. Klimek-Szczykutowicz M. Ekiert H. (2024). “ Elicitation as an effective biotechnological strategy for high production of bioactive secondary metabolites in plant *in vitro* cultures,” in Plant Specialized Metabolites: Phytochemistry, Ecology and Biotechnology ( Springer Nature, Switzerland), 1–48.

[B60] Talukder P. Chanda S. Sinha B. (2025). Boosting biotic stress resistance in *Solanum melongena* L.: the role of exogenous chlorogenic acid in enhancing secondary metabolite production. Appl. Biochem. Biotechnol. 197, 1–24. doi: 10.1007/s12010-025-05194-4, PMID: 39946059

[B61] Thiruvengadam R. Venkidasamy B. Easwaran M. Chi H. Y. Thiruvengadam M. Kim S. H. (2024). Dynamic interplay of reactive oxygen and nitrogen species (ROS and RNS) in plant resilience: unveiling the signaling pathways and metabolic responses to biotic and abiotic stresses. Plant Cell Rep. 43, 198. doi: 10.1007/s00299-024-03281-0, PMID: 39023775

[B62] Wang X. Ge M. He X. (2025). Effect of green synthesized Fe_3_O_4_NP priming on alfalfa seed germination under drought stress. Plants 14, 1236. doi: 10.3390/plants14081236, PMID: 40284124 PMC12030557

[B63] Wu Z. Hu Y. Hao R. Li R. Lu X. Itale M. W. . (2025). Research progress of genomics applications in secondary metabolites of medicinal plants: a case study in safflower. Int. J. Mol. Sci. 26, 3867. doi: 10.3390/ijms26083867, PMID: 40332590 PMC12027854

[B64] Xie M. Li F. Li Y. Qian K. Liang Y. Lei B. . (2025). Iron-doped carbon dots nanozyme scavenged reactive oxygen species system for inhibiting effectively the uptake of arsenic in lettuce. Chem. Engin. J. 506, 159956. doi: 10.1016/j.cej.2025.159956

[B65] Xu M. Xu D. (2024). “ Advanced systems and bioreactors for large-scale secondary metabolite production in medicinal plants using suspension cultured cells,” in Biosynthesis of Natural Products in Plants: Bioengineering in Post-Genomics Era ( Springer Nature, Singapore), 293–313.

[B66] Yang L. Chen H. Zhu S. Zhao S. Huang S. Cheng D. . (2024). Pectin-coated iron-based metal–organic framework nanoparticles for enhanced foliar adhesion and targeted delivery of fungicides. ACS Nano 18, 6533–6549. doi: 10.1021/acsnano.3c12352, PMID: 38355215

[B67] Yang T. Li H. Tai Y. Dong C. Cheng X. Xia E. . (2020). Transcriptional regulation of amino acid metabolism in response to nitrogen deficiency and nitrogen forms in tea plant root (*Camellia sinensis* L.). Sci. Rep. 10, 6868. doi: 10.1038/s41598-020-63835-6, PMID: 32321966 PMC7176667

[B68] Yousaf R. Khan M. A. Raza A. Ambreen H. Darwish H. Noureldeen A. (2025). Iron oxide nanoparticles and light intensity modulate biomass, antioxidant capacity and anti-leishmanial activity in callus cultures of *Artemisia scoparia*. Plant Cell Tissue Organ Cult. 160, 27. doi: 10.1007/s11240-025-02972-0

[B69] Zhang Y. Li L. Dai H. Kong X. Rahman M. Zhang B. . (2025). Iron oxide nanoparticles (FeO-NPs) mitigate salt stress in peanut seedlings by enhancing photosynthesis, osmoregulation, and antioxidant activity. Plant Physiol. Biochem. 110, 206. doi: 10.1016/j.plaphy.2025.110206, PMID: 40614542

[B70] Zhao B. Wang X. Liu H. Lv C. Lu J. (2020). Structural characterization and antioxidant activity of oligosaccharides from *Panax ginseng* C. A. Meyer. Int. J. Biol. Macromol. 150, 737–745. doi: 10.1016/j.ijbiomac.2020.02.016, PMID: 32027898

