# Supplementary material for: Nano-elicitation strategy to improve specialized metabolite pathways in plant cell suspension culture
Source: Front Plant Sci. 2025 Nov 28;16:1679901. doi: 10.3389/fpls.2025.1679901 (PMC12698641; doi:10.3389/fpls.2025.1679901)

**Supplementary Materials**

**Figure S1:** Alteration in expression of amino acid under control (CK) and nanoparticles (NPs) treated cells. * = *p* < 0.05, ** *p* < 0.01, represent statistical significance levels from a t-test.


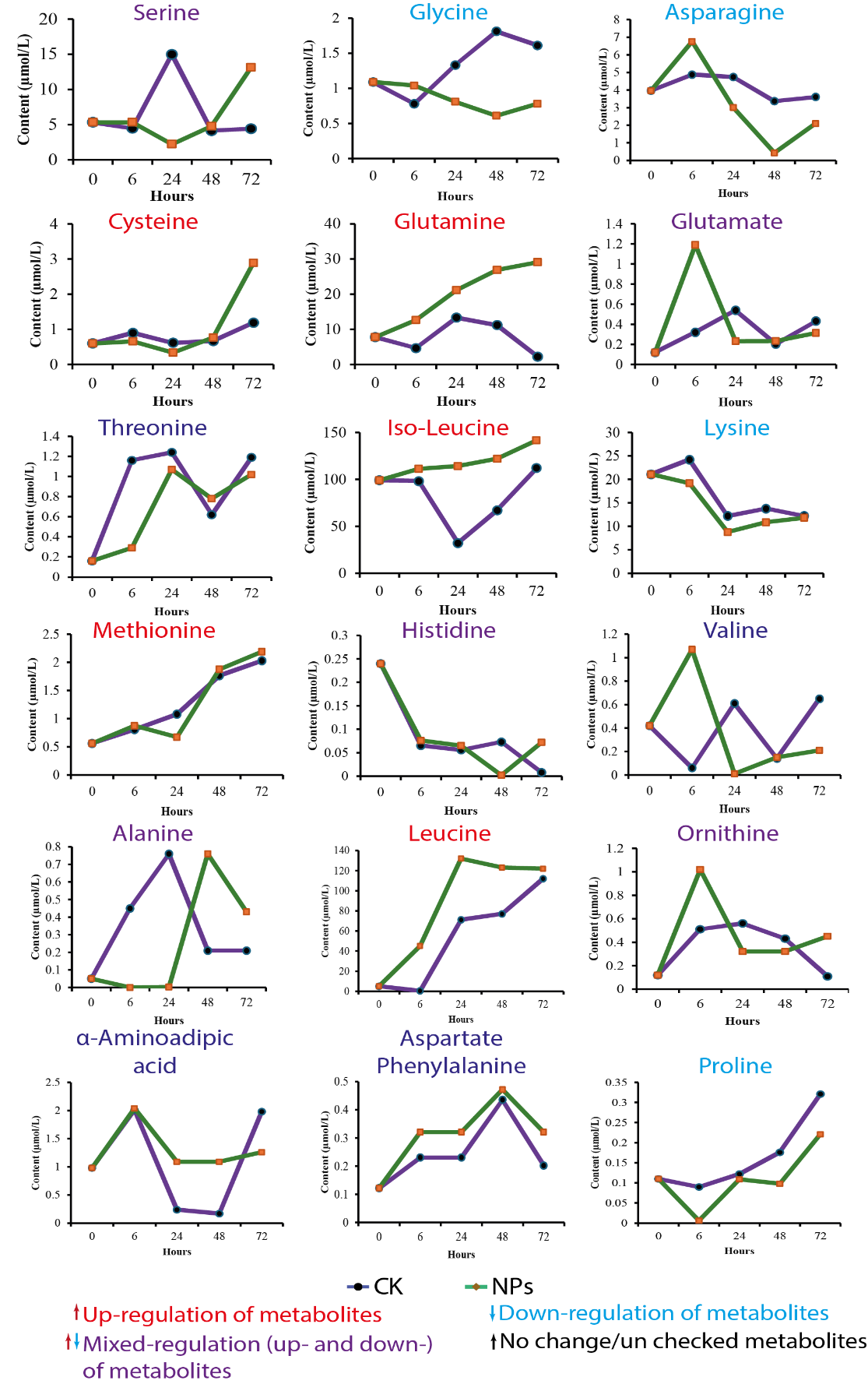

Supplement: Supplementary file 1 [file Table1.docx]
